# An improved hybrid whale optimization algorithm for global optimization and engineering design problems

**DOI:** 10.7717/peerj-cs.1557

**Published:** 2023-11-09

**Authors:** Abolfazl Rahimnejad, Ebrahim Akbari, Seyedali Mirjalili, Stephen Andrew Gadsden, Pavel Trojovský, Eva Trojovská

**Affiliations:** 1 Department of Mechanical Engineering, McMaster University, Hamilton, Canada; 2 Department of Mathematics, University of Hradec Králové, Hradec Králové, Czech Republic; 3 Centre for Artificial Intelligence Research and Optimisation, Torrens University Australia, Adelaide, Australia; 4 Yonsei Frontier Lab, Yonsei University, Seoul, South Korea

**Keywords:** Differential evolution algorithm, Friedman test, Metaheuristic optimization, Pbest-guided algorithm, Statistical tests, Whale optimization algorithm, Wilcoxon signed-rank test

## Abstract

The whale optimization algorithm (WOA) is a widely used metaheuristic optimization approach with applications in various scientific and industrial domains. However, WOA has a limitation of relying solely on the best solution to guide the population in subsequent iterations, overlooking the valuable information embedded in other candidate solutions. To address this limitation, we propose a novel and improved variant called Pbest-guided differential WOA (PDWOA). PDWOA combines the strengths of WOA, particle swarm optimizer (PSO), and differential evolution (DE) algorithms to overcome these shortcomings. In this study, we conduct a comprehensive evaluation of the proposed PDWOA algorithm on both benchmark and real-world optimization problems. The benchmark tests comprise 30-dimensional functions from CEC 2014 Test Functions, while the real-world problems include pressure vessel optimal design, tension/compression spring optimal design, and welded beam optimal design. We present the simulation results, including the outcomes of non-parametric statistical tests including the Wilcoxon signed-rank test and the Friedman test, which validate the performance improvements achieved by PDWOA over other algorithms. The results of our evaluation demonstrate the superiority of PDWOA compared to recent methods, including the original WOA. These findings provide valuable insights into the effectiveness of the proposed hybrid WOA algorithm. Furthermore, we offer recommendations for future research to further enhance its performance and open new avenues for exploration in the field of optimization algorithms. The MATLAB Codes of FISA are publicly available at https://github.com/ebrahimakbary/PDWOA.

## Introduction

As optimization problems in various disciplines become increasingly challenging, it becomes apparent that classical optimization methods suffer from limitations. These limitations include convergence to local optima, requirements of differentiability and continuity, and high computational burdens. Consequently, there is a growing need to develop more robust tools for optimal problem-solving. In recent years, metaheuristic methods, such as particle swarm optimization (PSO) (Kennedy & Eberhart, 1995) and genetic algorithm (GA) (Holland, 1992), have gained popularity and success in solving optimization problems. Various metaheuristic methods are still being proposed such as the termite life cycle optimizer (TLCO) (Minh et al., 2023b; Minh et al., 2023a), K-means optimizer (KO) (Minh et al., 2022), planet optimization algorithm (POA) (Sang-To et al., 2022), a combination of artificial neural network (ANN) and balancing composite motion optimization (BCMO) (Tran et al., 2023), and the new movement strategy of cuckoo search (NMS-CS) (Cuong-Le et al., 2021).

Researchers tend to utilize metaheuristic methods for optimization problems due to their derivative-free formulation and their ability to escape local optima and find global optima. However, it is important to consider the No Free Lunch theorem (Wolpert & Macready, 1997), which suggests that no single optimization algorithm performs best for all problems. Therefore, there is a need to explore and develop new metaheuristic algorithms that are specifically designed to address the challenges of different optimization problems.

The whale optimization algorithm (WOA) is a recent metaheuristic method suggested by Mirjalili & Lewis (2016), inspired by the hunting strategy of humpback whales. WOA has gained significant attention from engineers, designers, and researchers worldwide for its effectiveness in optimizing various problems. However, the original WOA formulation has a limitation: it only considers the best solution from each iteration, neglecting valuable information from other individuals and their best positions. This limitation can hinder the algorithm’s overall optimization performance.

To address this drawback, our proposed approach introduces an enhanced version of WOA called the Pbest-guided differential Whale Optimization Algorithm (PDWOA). PDWOA incorporates efficient features from PSO and differential evolution (DE) algorithms (Storn & Price, 1997) to improve the algorithm’s ability to avoid local optima and achieve global optima, particularly in shifted optimization problems. In addition, two non-parametric statistical tests, including the Wilcoxon signed-rank test and the Friedman test (Derrac et al., 2011; Buch, Trivedi & Jangir, 2017; Ghasemi et al., 2023), are employed to validate the performance improvements achieved by PDWOA over the original WOA.

The contributions of this study are outlined as follows:

1. Overview and analysis of the Whale Optimization Algorithm (WOA) to understand its functionality and limitations, particularly in complex real-world problems.
2. Development of a new enhanced version of WOA known as the Pbest-guided differential Whale Optimization Algorithm (PDWOA) to address the identified limitations of the original algorithm.
3. Evaluation of the performance of PDWOA compared to the original WOA through experiments on 30 shifted test functions from CEC2014. The results demonstrate the efficiency of PDWOA in obtaining optimal solutions. Statistical tests, such as the Wilcoxon signed-rank test and the Friedman test, are employed to validate the performance improvements.
4. Application of PDWOA to solve three real-world engineering problems, providing practical validation of its optimization performance in real-world scenarios.
5. Discussion of potential future improvements by exploring the integration of models from other powerful optimization algorithms, aiming to expand the range of problems that can be accurately solved by the proposed algorithm.

The remaining sections of this paper are organized as follows. The “Related Work” section provides an overview of the related work in the field. “WOA” presents a brief introduction to the WOA. The “Challenges and Enhanced Hybrid Version of WOA” section discusses the main drawbacks of WOA and proposes the Pbest-guided differential WOA (PDWOA) by incorporating efficient features of PSO and DE algorithms. The “Simulation Results” section presents the simulation results, where extensive experiments are conducted to evaluate the performance of PDWOA, including the statistical tests. “Discussion and Future Studies” discusses the results and provides potential areas for future studies. Finally, the paper is concluded in the “Conclusion” section.

## Related Works

A comprehensive overview of the applications of WOA, including various improvements, has been presented in Gharehchopogh & Gholizadeh (2019). Some notable examples of these improvements include the use of WOA for detecting weak signals in rotating (He et al., 2019), analyzing clinical data of anaemic pregnant (Saidala & Devarakonda, 2017), scheduling tasks in cloud computing (Sreenu & Sreelatha, 2017), and suppressing sidelobe in multiple input and multiple output radar systems (Yuan et al., 2018). Additionally, Mohammadi & Mehdizadeh (2020) proposed a novel hybrid model that combines support vector regression with WOA for the daily estimation of reference evapotranspiration, demonstrating superior performance compared to support vector regression-only models.

Qais, Hasanien & Alghuwainem (2020a) proposed a new enhanced version of WOA, called EWOA, specifically designed for maximizing power extraction from variable-speed wind generators (VSWGs). Instead of using the parameters suggested in the original WOA, EWOA incorporates a cosine function to control the searching and encircling behavior. Wang & Chen (2020) proposed a novel approach for medical diagnosis by improving a support vector machine (SVM) using chaotic WOA with multiple swarms (CMWOA). Their technique exhibited excellent performance in terms of avoiding local optima and achieving fast convergence. Cao et al. (2020) incorporated chaos theory to enhance the exploration ability and convergence characteristics of WOA, resulting in the development of a new chaotic-based improved version called CIWOA. This approach was specifically applied to achieve efficient terminal voltage control for proton exchange membrane fuel cells (PEMFCs).

Akyol & Alatas (2020) applied WOA and social impact theory based optimization for sentiment classification in online social media. Furthermore, Zeng et al. (2021) proposed a competitive mechanism enhanced WOA (CMWOA) for effectively addressing multi-objective optimization problems. Qais, Hasanien & Alghuwainem (2020b) introduced a novel design of Sugeno fuzzy logic controllers (FLCs) based on WOA (WOA-FLCs) to enhance the low voltage ride-through of VSWGs, resulting in improved time response characteristics surpassing those obtained by GA and grey wolf optimizer (GWO). Jain, Katarya & Sachdeva (2020) employed a novel social network-based WOA (SNWOA) to identify opinion leaders in social networks. Rosyadi, Penangsang & Soeprijanto (2017) applied the WOA to determine the optimal placement and size of filters in distribution systems. Chen, Li & Yang (2020) utilized chaos mechanism and quasi-opposition to enhance the convergence speed of WOA and mitigate the issue of local optima when solving large-scale problems. Liu et al. (2020) proposed the utilization of WOA for evaluating the resilience of regional flood disasters, demonstrating improved generalization performance and remarkable stability. Wang et al. (2019) introduced an opposition-based variant of WOA for tackling multi-objective optimization problems.

Srivastava et al. (2018) utilized WOA to estimate the parameters of a permanent magnet synchronous motor. An improved version of WOA optimizer was suggested in Abdel-Basset, Mohamed & Mirjalili (2021), which comprises three modifications compared to the original WOA. Firstly, the dynamic distance control factor was used rather than a fixed one. Secondly, a certain probability was used to achieve a compromise between movement towards the best solution and its opposite for escaping from local optimal solutions. Finally, Nelder–Mead was used along with the Pareto archived evolution strategy (PAES) to further improve WOA. Authors of Mahdad (2018) solved the optimal power flow (OPF) problem utilizing a new partitioning whale algorithm.

In Chen et al. (2020), an improved WOA named RDWOA was suggested for improving the convergence and global optimization performance of WOA in solving multi-dimensional problems. The improvement included two schemes, random spare or random replacement and double adaptive weight, which were used for advancing the convergence, exploration at the initial phases, and exploitation at subsequent phases. The proposed strategies considerably increased the convergence speed and the optimization performance of WOA. The efficiency of RDWOA was proved by utilizing typical benchmarks and engineering problems.

Trivedi et al. (2016) applied WOA to solve emission constraint environmental economic dispatch problems. Tu et al. (2021) proposed another enhanced variant of WOA to improve its convergence performance and prevent being trapped in local optimal solutions. The enhancement employs a new communication mechanism (CM) for improving the global optimization performance and biogeography-based optimization (BBO) to compromise between the exploring and exploiting performances. The effectiveness of BBO was confirmed using benchmark and engineering problems.

Abdel-Basset, Abdle-Fatah & Sangaiah (2018) proposed an enhanced version of WOA that incorporates Lévy flight (LF) for problem-solving in the cloud computing environment. Mafarja & Mirjalili (2017) proposed a hybrid approach that combines WOA with simulated annealing for feature selection. In Nazari-Heris et al. (2017), the optimal generations of combined heat and power units were determined using WOA. Guo et al. (2020) augmented WOA by incorporating adaptive social learning (ASL) and wavelet mutation. At first, a novel exploration probability was formulated for improving the performance of WOA. Then, an ASL strategy was utilized for constructing the adaptive social network (ASN) of the WOA population, to enhance its diverseness. Finally, the suggested procedure was augmented using the Morlet wavelet mutation strategy. WOA was proposed in Reddy, Reddy & Manohar (2017) for the solution of optimally identifying the size of renewable energy resources.

In Samadianfard et al. (2020) a hybridization of the multi-layer perceptron (MLP) neural network and WOA was roposed for wind speed forecasting. Content-based image retrieval was solved in Aziz, Ewees & Hassanien (2018) using multi-objective WOA (MOWOA) algorithm. In Wu et al. (2018), the path planning problem for solar-powered UAV in urban environment was solved using WOA enhanced with adaptive chaos-Gaussian switching solving strategy and coordinated decision-making mechanism. In Hou et al. (2020), a hybrid of quantum simultaneous WOA (QSWOA) and a multi-objective economic model predictive control (MOEMPC) was proposed for controlling gas turbines. A new improved opposition-based WOA (IOWOA) was used for estimating the parameters of solar cells diode models (Abd Elaziz & Oliva, 2018). Binary WOA was utilized in Eid (2018) to deal with feature selection problems. In Liu, Yao & Li (2020) a hybridization of LF-augmented WOA and DE was suggested for dealing with the job shop scheduling problem (JSSP), where, LF and DE are used for improving the exploration and exploitation performances, respectively. Data clustering based on WOA was proposed in Canayaz & Özdağ (2017). Qiao et al. (2020) employed a novel improved variant of WOA called IWOA for short-term natural gas consumption forecasting. Khalilpourazari, Pasandideh & Ghodratnama (2018) proposed the utilization of Whale Optimization Algorithm (WOA) and Water Cycle Algorithm (WCA) for programming a multi-item economic order quantity model. Pham et al. (2020) proposed the utilization of WOA for the optimal allocation of resources in wireless networks.

## WOA

The mathematical model of the original WOA, inspired by the hunting strategy of humpback whales, which consists of the stages of surrounding prey, bubble-net hunting maneuver, and search for prey, is briefly discussed in this section, Mirjalili & Lewis (2016).

### Encircling prey

During the encircling prey stage, the WOA algorithm emulates the ability of humpback whales to recognize the prey’s position and encircle them. In each iteration, the best solution, acting as the leader, is considered the target prey. The behavior is defined in Eqs. (1)–(3):

(1) \begin{eqnarray*}X^{\rightarrow } \left( t+1 \right) ={X^{\rightarrow }}_{Leader} \left( t \right) -A^{\rightarrow }\odot D^{\rightarrow }\end{eqnarray*}

(2) \begin{eqnarray*}A^{\rightarrow }=2a.r^{\rightarrow }-a\end{eqnarray*}

(3) \begin{eqnarray*}C^{\rightarrow }=2.r^{\rightarrow }\end{eqnarray*}

where, *t* represents the iteration number, $A^{\rightarrow }$ and $C^{\rightarrow }$ are the coefficient vectors of WOA, ${X^{\rightarrow }}_{Leader}$ denotes the position vector of the best solution found so far, and $X^{\rightarrow }$ represents the position vector of each member in the algorithm population. Furthermore, the ⊙ sign denotes the element-wise multiplication. Notably, to enhance the performance of WOA, the value of *a* linearly declines from 2 to zero during the iterations, and $r^{\rightarrow }$ is a vector of uniformly distributed random numbers between zero and one.

### Exploitation stage: bubble-net attacking maneuver

The bubble-net attacking maneuver, inspired by the hunting behavior of humpback whales, is modeled using two strategies:

**1. Declining surrounding strategy:** This strategy is achieved by reducing the value of *a* in Eq. (2). Notably, the range of variation in vector $A^{\rightarrow }$ is directly proportional to *a*, where $A^{\rightarrow }$ consists of randomly generated values between - *a* and *a*.

**2. Spiral position update:** The whale’s displacement towards the prey’s position, simulating the spiral motion of humpback whales, is formulated as Eq. (4):

(4) \begin{eqnarray*}X^{\rightarrow } \left( t+1 \right) ={X^{\rightarrow }}_{Leader} \left( t \right) +{D}^{{^{\prime}}}^{\rightarrow }.{e}^{B.L}.Cos \left( 2\pi L \right) \end{eqnarray*}

where ${D}^{{^{\prime}}}^{\rightarrow }= \left\vert {X^{\rightarrow }}_{Leader}-X^{\rightarrow } \right\vert $ denotes how far is the *i*th whale from the prey, *B* is a constant that describes the logarithmic spiral motion, and *L* is a random value between −1 and 1.

It is important to mention that the selection between the declining surrounding strategy and the spiral position update is equally probable.

### Search for prey

The search for prey, representing the exploration stage of WOA, can be achieved by adjusting the vector $A^{\rightarrow }$. This mechanism facilitates a global search by setting the absolute value of the vector $A^{\rightarrow }$ to ${|}A^{\rightarrow }{|}\gt 1$. Mathematically, this stage can be formulated as Eqs. (5) and (6):

(5) \begin{eqnarray*}D^{\rightarrow }= \left\vert C^{\rightarrow }\odot {X^{\rightarrow }}_{rand}-X^{\rightarrow } \right\vert \end{eqnarray*}

(6) \begin{eqnarray*}X^{\rightarrow } \left( t+1 \right) ={X^{\rightarrow }}_{rand}-A^{\rightarrow }\odot D^{\rightarrow }\end{eqnarray*}

where ${X^{\rightarrow }}_{rand}$ denotes the position vector of an indiscriminately chosen solution. It is important to note that the WOA method relies on two main parameters, $A^{\rightarrow }$ and $C^{\rightarrow }$, which need to be tuned.

## Challenges and Enhanced Hybrid Version of WOA

In the current section, the challenges of WOA are discussed, followed by the introduction of a novel improved version of WOA to address those challenges.

### Challenges of WOA

In practical applications, we encounter optimization problems with diverse behaviors and levels of complexity. Therefore, researchers strive to find an algorithm that is robust, requires minimal parameter tuning, and offers simplicity and fast convergence speed (Talbi, 2009). Real-world problems often involve shifted functions, where the global optimal solutions do not reside at the origin of coordinates and vary across dimensions. It is well-documented in the literature that many algorithms exhibit reduced performance for shifted functions (Liang, Qu & Suganthan, 2013), which necessitates appropriate modifications.

**Figure 1:**
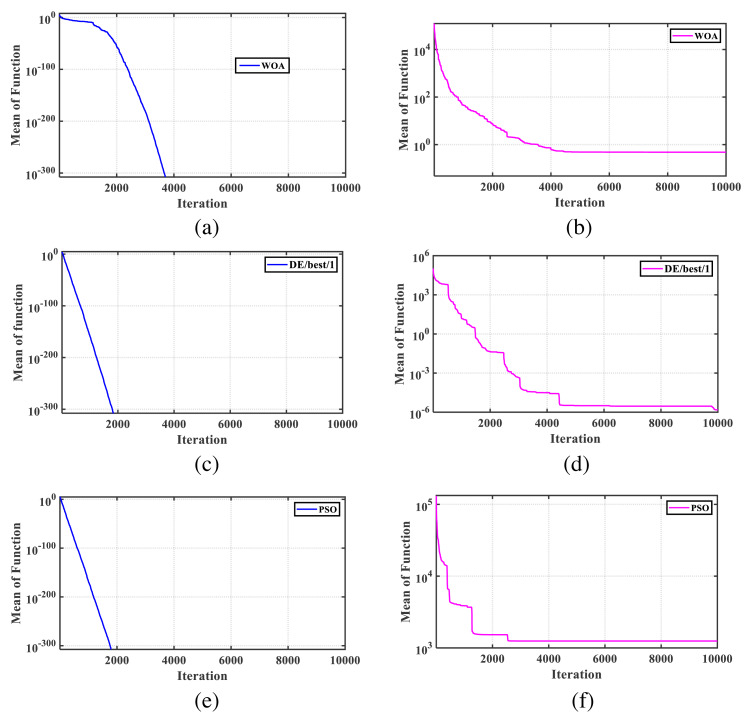
The convergence characteristics for the standard test functions. (A) Sphere function solution obtained by WOA; (B) shifted sphere function solution obtained by WOA (mean is 4.786e−1); (C) sphere function solution obtained by DE/best/1; (D) shifted sphere function solution obtained by DE/best/1 (mean is 1.510e−06); (E) sphere function solution obtained by PSO; (F) shifted sphere function solution obtained by PSO (mean is 1.247e3).

To investigate this issue with WOA, we conducted experiments using the conventional model of the sphere function (Mirjalili & Lewis, 2016) and its shifted counterpart, known as the Shifted Sphere Function (Suganthan et al., 2005). We aimed to determine the optimal solutions for these functions with 30 dimensions using WOA, PSO, and DE methods. Each function was independently evaluated in 25 runs, with 300,000 function evaluations (Suganthan et al., 2005) and a population size of 30 for the algorithm. The mean values obtained by WOA for the optimal response of the traditional sphere function and the shifted sphere function were 0 and 0.478, respectively. Figure 1 illustrates the convergence characteristics of WOA, DE/best/1, and PSO algorithms for both functions. It is worth noting that all algorithm parameters were set according to the recommendations in the original codes, leading to improved average performance across a wide range of problems. From the figure, it is evident that the original WOA exhibits reduced performance for shifted functions. Therefore, it is crucial to either tune the key controlling parameters or modify the WOA formulation to enhance its efficiency in solving a wider range of engineering and real-world problems.

Another issue with the original WOA is that it only stores the best solution among the entire population in each iteration. In contrast, algorithms like particle swarm optimization (PSO) store the personal best position (Pbest) for each member in each iteration, which enables directing the population members to avoid local optima. Therefore, an enhanced hybrid model of WOA can be developed by leveraging the advantageous features of other algorithms as an auxiliary operator. In this study, we present a new efficient hybrid variant of WOA that incorporates the formulations of PSO (Eberhart & Kennedy, 1995) and differential evolution (DE) (Storn & Price, 1997). This hybrid variant will be discussed in detail in the next section.

### Pbest-guided differential WOA

The storage of only the best solution in WOA, similar to GA, is identified as a fundamental weakness of the algorithm based on our investigation. This limitation arises from eliminating many candidate solutions in each iteration, which could potentially be useful in subsequent iterations and enhance the algorithm’s optimization capability, as observed in DE and PSO algorithms. Consequently, we can leverage the models/formulations of basic DE, PSO, and their advanced variants, which have gained significant popularity in recent years, to enhance WOA’s performance in locating the global optimum of real-world optimization problems.

***PSO-based Modification****:* The first modification proposed in this study involves storing the personal best(*Pbest*) position of each member in each iteration, denoted by →*Xpbest* > 1, similar to the PSO algorithm. With this Pbest-guided modification, the search equations can be rewritten as Eqs. (7)–(14):

(7) \begin{eqnarray*}A^{\rightarrow }=2a.r^{\rightarrow }-a\end{eqnarray*}

(8) \begin{eqnarray*}C^{\rightarrow }=2r^{\rightarrow }\end{eqnarray*}

(9) \begin{eqnarray*}D^{\rightarrow }= \left\vert C^{\rightarrow }\odot {X^{\rightarrow }}_{Leader} \left( t \right) -Xpbest^{\rightarrow } \left( t \right) \right\vert \end{eqnarray*}

(10) \begin{eqnarray*}X^{\rightarrow } \left( t+1 \right) ={X^{\rightarrow }}_{Leader} \left( t \right) -A^{\rightarrow }\odot D^{\rightarrow }\end{eqnarray*}

(11) \begin{eqnarray*}{D}^{{^{\prime\prime}}}^{\rightarrow }= \left\vert C^{\rightarrow }\odot {X^{\rightarrow }}_{rand}-Xpbest^{\rightarrow } \right\vert \end{eqnarray*}

(12) \begin{eqnarray*}X^{\rightarrow } \left( t+1 \right) ={X^{\rightarrow }}_{rand}-A^{\rightarrow }\odot {D^{\rightarrow }}^{{^{\prime\prime}}}\end{eqnarray*}

(13) \begin{eqnarray*}{D}^{{^{\prime}}}^{\rightarrow }= \left\vert {X^{\rightarrow }}_{Leader}-Xpbest^{\rightarrow } \right\vert \end{eqnarray*}

(14) \begin{eqnarray*}X^{\rightarrow } \left( t+1 \right) ={X^{\rightarrow }}_{Leader} \left( t \right) +{D}^{{^{\prime}}}^{\rightarrow }.{e}^{BL}.Cos \left( 2\pi L \right) \end{eqnarray*}

where Eqs. (9) and (10) denote the encircling prey phase, Eqs. (11) and (12) model the search for prey phase, and Eqs. (13) and (14) demonstrate the spiral position update. Note that in the proposed algorithm, the same as PSO, for each member of the population in each iteration, $Xpbest^{\rightarrow }$ is updated for each individual as Eq. (15):

(15) \begin{eqnarray*}Xpbest^{\rightarrow } \left( t+1 \right) = \left\{ \begin{array}{@{}l@{}} \displaystyle X^{\rightarrow } \left( t+1 \right) ;\begin{array}{@{}l@{}} \displaystyle \end{array}if\,f \left( X^{\rightarrow } \left( t+1 \right) \right) \leq f \left( Xpbest^{\rightarrow } \left( t \right) \,\, \right) \\ \displaystyle Xpbest^{\rightarrow } \left( t \right) \,;\begin{array}{@{}l@{}} \displaystyle \end{array}else \end{array} \right. \end{eqnarray*}

***DE-based modification:*** In the DE-based modification, we incorporate the best position found by each individual in all previous iterations. This enables us to leverage the mutations proposed in the DE algorithm to effectively enhance the original WOA. Therefore, in the second stage of the modification, a mutation phase, as defined in Eq. (16), is added to the formulation of WOA immediately after the main phases of the algorithm:

(16) \begin{eqnarray*}\begin{array}{@{}l@{}} \displaystyle V^{\rightarrow } \left( t \right) =Xpbest^{\rightarrow } \left( t \right) +rand1 \left( {X^{\rightarrow }}_{Leader} \left( t \right) -Xpbest^{\rightarrow } \left( t \right) \right) \\ \displaystyle  +rand2 \left( Xpbes{t}_{r1}^{\rightarrow } \left( t \right) -Xpbes{t}_{r2}^{\rightarrow } \left( t \right) \right) \end{array}\end{eqnarray*}

where $Xpbes{t}_{r1}^{\rightarrow }$ and $Xpbes{t}_{r2}^{\rightarrow }$ are the personal best positions of two solutions randomly chosen from the population for updating each solution. Similarly, *rand*1 and *rand*2 are random vectors with dimensions equal to *D* (problem’s dimension), where the elements’ values range between 0 and 1. Subsequently, a random variable *randj* is generated for each dimension *j* of each solution, leading to Eq. (17):

(17) \begin{eqnarray*}{X}_{j}^{\rightarrow } \left( t+1 \right) = \left\{ \begin{array}{@{}l@{}} \displaystyle {V^{\rightarrow }}_{j} \left( t \right)  if\,randj\gt Cr\\ \displaystyle \\ \displaystyle {X}_{j}^{\rightarrow } \left( t \right)  \text{otherwise} \end{array}. \right. \end{eqnarray*}

Here, *Cr* represents a control parameter, similar to the crossover rate used in evolutionary algorithms.

**Figure 2:**
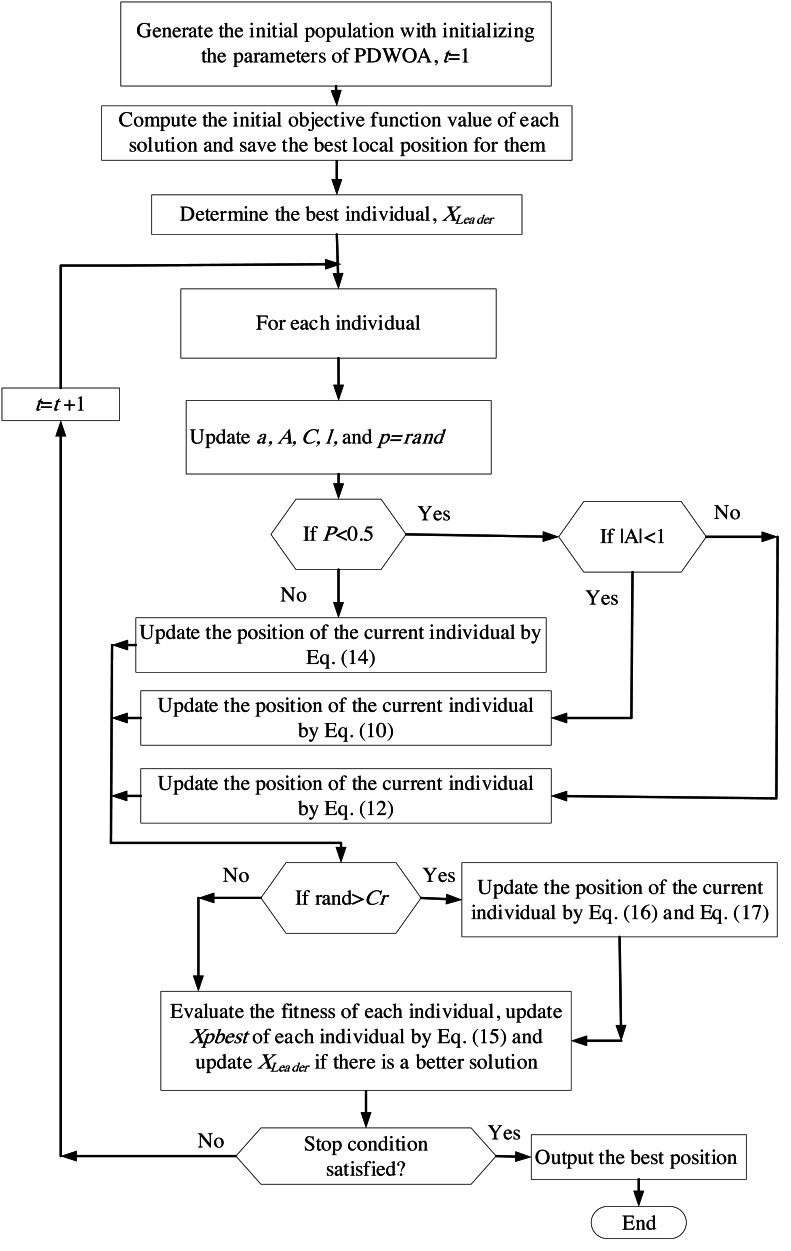
Flowchart of PDWOA.

Finally, we want to emphasize that we employed the penalty method, a widely-used approach for addressing constraints in constrained optimization problems, in our research. The penalty method utilizes penalty functions to guide the optimization algorithm towards feasible solutions while penalizing infeasible solutions. To provide a visual representation of the proposed approach, we have included a flowchart of the Pbest-guided differential Whale Optimization Algorithm (PDWOA) in Fig. 2.

## Simulation Results

The verification of the effectiveness of PDWOA is achieved using two sets of experiments, firstly it was used for solving CEC 2014 Test Functions (Liang, Qu & Suganthan, 2013), and then it is exploited for solving three engineering problems.

### Solving CEC 2014 test functions using PDWOA

In order to compare the performance of PDWOA with that of the original WOA, 30 test functions with 30 dimensions have been selected from CEC 2014 Test Functions (Liang, Qu & Suganthan, 2013). These functions include unimodal (F1-F3), multimodal (F4-F16), hybrid (F17-F22), and composition (F23-F30) functions. For both algorithms, we consider a population number equal to 30 and iteration numbers equal to 10,000, *i.e.,* the number of function evaluations (NFEs) done by each algorithm (for each test function) is 300,000. To find the optimal solution of each function, 25 separate runs have been executed for each algorithm and then, statistical analysis has been performed on the results.

A comparative study between DE, PSO, the original WOA, and the proposed PDWOA with three different *Cr* settings, *i.e.,* a random value and the fixed values of 0.1 and 0.9, is presented in Table 1. In this table, the terms “Mean” and “Std”. represent the average value and standard deviation, respectively, of the results obtained from 25 independent runs for optimizing each function using each algorithm. The term “Rank” indicates the ranking of the algorithm’s Mean index, reflecting its effectiveness in optimizing the considered function. Additionally, “NB” represents the number of functions for which the algorithm achieves the best Mean index, while “MR” represents the mean of the Rank indices of the algorithm across all functions. It is evident from the table that the proposed algorithms, with two different *Cr* tunings, outperform the original algorithm significantly. Specifically, the PDWOA with *Cr* set to a random value and 0.1 surpasses the performance of the original WOA for 21 and 24 shifted test functions, respectively. Notably, even in cases where the suggested algorithm exhibits worse performance, the resulting outcomes do not deviate significantly from those obtained by the original WOA.

**Table 1:** Summary of the results of DE, PSO, WOA and different variants of PDWOA for CEC 2014 test functions.

Function	DE/best/1	PSO	WOA	PDWOA/*Cr*= rand	PDWOA/*Cr*= 0.1	PDWOA/*Cr*= 0.9
	Mean Std. Rank	Mean Std. Rank	Mean Std. Rank	Mean Std. Rank	Mean Std. Rank	Mean Std. Rank
F1	1.11E+08 4.23E+07 6	4.38E+07 1.81E+07 5	3.39E+07 1.96E+07 4	3.15E+06 1.75E+06 1	3.31E+06 2.11E+06 2	4.08E+06 2.92E+06 3
F2	1.28E+10 6.28E+09 6	9.83E+08 5.36E+08 5	3.43E+06 1.38E+06 4	1.42E+04 1.32E+04 1	2.45E+04 1.43E+04 3	1.46E+04 1.26E+04 2
F3	9.32E+04 5.80E+04 6	2.84E+04 1.40E+04 4	4.50E+04 3.03E+04 5	3.51E+03 3.56E+03 2	1.59E+03 2.11E+03 1	1.13E+04 1.85E+04 3
F4	1.27E+03 6.92E+02 6	2.61E+02 8.36E+01 5	1.97E+02 4.93E+01 4	1.23E+02 3.19E+01 2	1.03E+02 4.01E+01 1	1.37E+02 4.45E+01 3
F5	2.09E+01 5.00E−02 6	2.04E+01 6.00E−02 5	2.02E+01 1.70E−01 3	2.02E+01 1.50E−01 2	2.03E+01 2.70E−01 4	2.02E+01 3.30E−01 1
F6	2.04E+01 3.53E+00 1	2.95E+01 2.70E+00 4	3.71E+01 4.11E+00 5	2.63E+01 3.33E+00 2	3.45E+01 4.44E+00 3	4.00E+01 1.86E+00 6
F7	1.33E+02 7.20E+01 6	1.50E+01 1.36E+01 5	1.03E+00 4.00E−02 4	9.00E−02 1.00E−01 1	1.80E−01 3.30E−01 3	1.00E−01 1.20E−01 2
F8	1.08E+02 3.04E+01 2	1.46E+02 2.85E+01 3	1.79E+02 2.34E+01 4	6.64E+01 1.68E+01 1	1.85E+02 2.82E+01 5	2.15E+02 6.27E+01 6
F9	1.76E+02 4.24E+01 1	1.78E+02 2.72E+01 2	2.29E+02 2.83E+01 5	2.05E+02 5.24E+01 3	2.22E+02 6.47E+01 4	2.38E+02 4.51E+01 6
F10	3.07E+03 4.54E+02 2	4.37E+03 6.39E+02 6	3.94E+03 1.05E+03 4	6.27E+02 4.05E+02 1	3.54E+03 7.29E+02 3	4.36E+03 4.04E+02 5
F11	3.15E+03 6.56E+02 1	4.90E+03 9.12E+02 6	4.77E+03 8.17E+02 5	4.11E+03 8.53E+02 2	4.48E+03 5.99E+02 4	4.47E+03 1.11E+03 3
F12	2.13E+00 1.06E+00 6	1.36E+00 3.90E−01 2	1.42E+00 6.10E−01 4	1.15E+00 6.70E−01 1	1.39E+00 6.60E−01 3	1.57E+00 3.80E−01 5
F13	3.03E+00 9.40E−01 6	5.40E−01 7.00E−02 3	5.20E−01 8.00E−02 2	5.10E−01 1.30E−01 1	6.30E−01 1.40E−01 5	5.60E−01 1.20E−01 4
F14	4.99E+01 3.52E+01 6	1.63E+00 2.84E+00 5	2.70E−01 4.00E−02 2	3.00E−01 5.00E−02 3	2.60E−01 5.00E−02 1	4.10E−01 3.30E−01 4
F15	7.41E+04 7.77E+04 6	1.05E+02 6.57E+01 3	7.88E+01 1.87E+01 2	4.55E+01 1.21E+01 1	5.26E+02 6.32E+02 4	7.92E+02 2.71E+02 5
F16	1.15E+01 4.00E−01 1	1.28E+01 5.60E−01 4	1.24E+01 4.60E−01 3	1.17E+01 4.00E−01 2	1.31E+01 3.30E−01 6	1.30E+01 5.80E−01 5
F17	3.65E+06 2.86E+06 5	1.14E+06 8.15E+05 4	5.02E+06 2.18E+06 6	7.82E+05 3.34E+05 3	6.39E+05 6.53E+05 1	7.74E+05 2.98E+05 2
F18	1.17E+06 2.57E+06 6	5.40E+03 2.45E+03 4	3.32E+04 7.93E+04 5	4.34E+03 4.79E+03 1	4.79E+03 6.86E+03 2	4.80E+03 6.01E+03 3
F19	7.42E+01 4.08E+01 5	3.23E+01 2.50E+01 2	5.66E+01 5.04E+01 3	2.39E+01 2.67E+01 1	6.72E+01 5.07E+01 4	8.91E+01 3.61E+01 6
F20	2.71E+04 3.00E+04 5	1.04E+04 5.33E+03 4	2.90E+04 2.30E+04 6	7.76E+03 4.60E+03 3	2.33E+03 1.82E+03 1	3.56E+03 2.78E+03 2
F21	7.40E+05 7.73E+05 6	1.13E+05 1.27E+05 1	9.29E+05 6.55E+05 5	4.87E+05 2.88E+05 2	5.23E+05 3.45E+05 3	5.55E+05 4.00E+05 4
F22	6.16E+02 1.54E+02 1	7.26E+02 1.21E+02 2	9.17E+02 3.09E+02 4	9.87E+02 1.88E+02 5	8.67E+02 3.04E+02 3	1.34E+03 2.83E+02 6
F23	3.99E+02 7.66E+01 6	3.40E+02 7.70E+00 5	3.34E+02 4.88E+00 4	3.15E+02 2.00E−02 1	3.15E+02 7.00E−02 2	3.16E+02 9.60E−01 3
F24	3.04E+02 2.66E+01 6	2.55E+02 7.98E+00 5	2.06E+02 4.44E+00 1	2.18E+02 1.12E+01 4	2.06E+02 5.36E+00 3	2.06E+02 2.38E+00 2
F25	2.24E+02 6.67E+00 6	2.22E+02 4.31E+00 4	2.18E+02 1.88E+01 2	2.23E+02 1.63E+01 5	2.09E+02 1.31E+01 1	2.21E+02 2.08E+01 3
F26	1.85E+02 4.57E+01 5	1.73E+02 4.93E+01 4	1.00E+02 1.10E−01 1	1.15E+02 3.76E+01 2	1.57E+02 1.13E+02 3	1.87E+02 1.08E+02 6
F27	9.76E+02 2.08E+02 3	9.04E+02 3.26E+02 2	1.20E+03 3.32E+02 5	1.11E+03 8.11E+01 4	7.95E+02 4.61E+02 1	1.32E+03 3.90E+02 6
F28	2.26E+03 5.47E+02 2	3.45E+03 1.67E+03 6	2.51E+03 6.25E+02 4	1.88E+03 8.69E+02 1	2.46E+03 1.21E+03 3	2.83E+03 1.38E+03 5
F29	3.17E+06 4.31E+06 1	2.09E+07 3.46E+07 4	5.21E+06 4.84E+06 2	7.54E+06 5.27E+06 3	3.27E+07 2.74E+07 5	3.79E+07 4.11E+07 6
F30	2.56E+05 1.79E+05 6	5.10E+04 7.03E+04 3	9.98E+04 5.98E+04 4	3.91E+03 1.91E+03 1	8.93E+03 3.30E+03 2	1.07E+05 1.76E+05 5
NB/MR	6/4.3667	1/4.2333	2/3.6667	7/2.8667	13/2.0	1/4.0667

**Figure 3:**
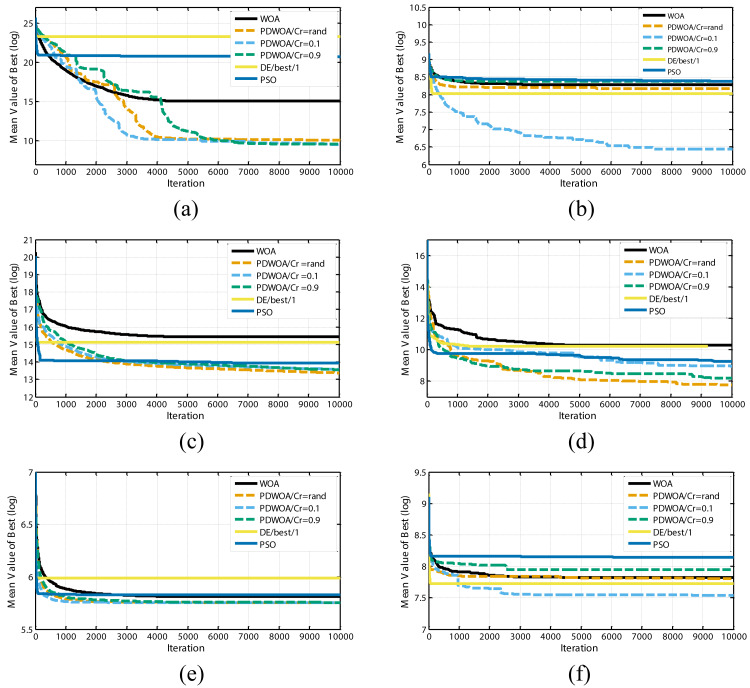
Convergence curves of optimization algorithms for; (A) F2; (B) F10; (C) F17; (D) F20; (E) F23 and (F) F28 shifted test functions.

It can be further seen from results in Table 1 the suggested PDWOA could attain results of a much higher quality for test functions F1, F2, F3, F7, F10, F17, F18, F20, and F30 compared to the original algorithm. Furthermore, the convergence characteristics of the algorithms for some of the test functions are depicted in Fig. 3, which confirms the higher performance and convergence rate of the suggested PDWOA.

Table 2 presents the average simulation time of 25 runs for each of the CEC 2014 test functions, with the aim of comparing the computational burden of the proposed PDWOA to that of the original WOA, PSO, and DE algorithms. It is important to note that, due to the small difference in computational burden between different versions of PDWOA, only the simulation times of PDWOA/Cr = rand are reported in this table. The results indicate that, for 29 and 25 out of 30 test functions, PDWOA has lower mean simulation times than DE and PSO algorithms, respectively. However, for 24 out of 30 test functions, PDWOA has higher mean simulation times than the original WOA. Nonetheless, the maximum increase in mean simulation times by using the proposed improved version of WOA is only about 8%, occurring for test function 1. This increase is not too high considering the degree of improvement in the final solutions.

**Table 2:** Mean simulation times (s) of 25 runs of different algorithms in solving each of the CEC 2014 test functions.

Function	DE/best/1	PSO	WOA	PDWOA/*Cr*= rand
F1	6.47	5.94	4.95	5.35
F2	5.79	5.29	4.41	4.73
F3	5.97	5.18	4.32	4.64
F4	5.92	5.06	4.29	4.62
F5	5.81	5.44	4.59	4.87
F6	30.22	29.1	30.83	30.79
F7	5.89	5.41	4.71	5.02
F8	5.54	5.06	4.18	4.48
F9	5.84	5.43	4.57	4.83
F10	6.7	6.06	5.42	5.7
F11	7.2	6.47	5.88	5.98
F12	10.8	10.03	9.47	9.63
F13	5.61	4.87	4.32	4.57
F14	5.66	5.07	4.39	4.53
F15	6.26	5.49	4.62	4.96
F16	5.99	5.25	4.68	4.98
F17	6.73	5.99	5.16	5.42
F18	6.03	5.59	4.65	4.88
F19	11.21	9.72	9.95	9.95
F20	6.32	5.51	4.67	4.97
F21	6.61	5.9	5.03	5.29
F22	6.88	6.1	5.5	5.7
F23	12.16	11.31	10.76	11.01
F24	10.11	9.23	8.64	8.75
F25	11.29	10.14	9.75	9.94
F26	40.57	36.66	39.06	38.5
F27	38.73	36.78	38.97	38.36
F28	14.18	12.95	12.73	12.7
F29	15.15	13.77	14.4	13.94
F30	11.01	10.1	9.67	9.79

Table 3 displays a comparative analysis of the performance of the selected variant of the proposed Pbest-guided differential Whale Optimization Algorithm (*i.e.,* PDWOA/Cr = rand) and several other state-of-the-art methods, including Arithmetic Optimization Algorithm (AOA) (Abualigah et al., 2021), Hierarchical Multi-swarm Cooperative TLBO (HMCTLBO) (Zou et al., 2017), Moth-Flame Optimization algorithm (MFO) (Mirjalili, 2015), Adaptive Weighted Particle Swarm Optimizer (AWPSO) (Liu et al., 2021), Gaussian bare-bones gradient-based optimization (GOMGBO) (Qiao et al., 2022), and Lévy flight Jaya Algorithm (LJA) (Iacca, dos Santos Junior & Veloso de Melo, 2021), for solving CEC 2014 test functions.

**Table 3:** Summary of the results of PDWOA and several state-of-the-art methods for CEC 2014 test functions.

Function	AOA	HMCTLBO	AWPSO	GOMGBO	MFO	LJA	PDWOA/*Cr*= rand
	Mean Std. -/+/=	Mean Std. -/+/=	Mean Std. -/+/=	Mean Std. -/+/=	Mean Std. -/+/=	Mean Std. -/+/=	Mean Std.
F1	3.026E+07 9.066E+06 –	2.659E+07 1.490E+07 –	1.356E+07 1.417E+07 –	3.238E+07 2.218E+07 –	7.59E+07 9.77E+07 –	6.31E+07 1.87E+07 –	3.15E+06 1.75E+06
F2	1.077E+07 1.500E+07 –	1.332E+06 9.362E+05 –	6.615E+07 1.141E+08 –	9.280E+07 1.260E+08 –	1.36E+10 8.42E+09 –	4.77E+09 6.03E+08 –	1.42E+04 1.32E+04
F3	2.730E+04 1.121E+04 –	1.303E+04 1.654E+03 –	2.669E+04 1.650E+04 –	1.994E+04 3.669E+03 –	8.99E+04 4.98E+04 –	6.91E+04 1.07E+04 –	3.51E+03 3.56E+03
F4	1.887E+02 1.036E+02 –	2.434E+02 1.483E+02 –	5.777E+02 5.774E+02 –	2.207E+02 5.991E+01 –	1.14E+03 1.13E+03 –	4.08E+02 5.38E+01 –	1.23E+02 3.19E+01
F5	2.06E+01 5.865E−02 –	2.084E+01 2.306E−01 –	2.061E+01 9.313E−02 –	2.041E+01 1.266E−01 –	2.04E+01 1.75E −01 –	2.09E+01 4.97E–02 –	2.02E+01 1.50E−01
F6	4.217E+01 1.739E+00 –	3.871E+01 2.091E+00 –	4.103E+01 1.144E+00 –	4.231E+01 1.126E+00 –	2.40E+01 3.33E+00 +	3.39E+01 1.29E+00 –	2.63E+01 3.33E+00
F7	8.071E−01 2.439E−01 –	6.374E−01 5.306E−01 –	9.228E−01 2.835E−01 –	5.078E−01 1.841E−01 –	1.17E+02 6.91E+01 –	1.58E+01 2.80E+00 –	9.00E−02 1.00E−01
F8	1.775E+02 2.291E+01 –	1.979E+02 5.195E+01 –	2.096E+02 1.096E+01 –	2.413E+02 6.580E+01 –	1.43E+02 3.81E+01 –	2.24E+02 9.93E+00 –	6.64E+01 1.68E+01
F9	2.346E+02 5.240E+01 –	2.933E+02 1.168E+02 –	2.188E+02 9.356E+01 –	1.952E+02 2.288E+01 +	2.23E+02 6.06E+01 –	2.61E+02 1.47E+01 –	2.05E+02 5.24E+01
F10	4.937E+03 9.675E+02 –	5.549E+03 5.726E+02 –	4.533E+03 9.972E+02 –	4.475E+03 3.306E+02 –	3.47E+03 8.85E+02 –	5.68E+03 3.95E+02 –	6.27E+02 4.05E+02
F11	5.683E+03 5.618E+02 –	5.416E+03 1.132E+03 –	4.937E+03 1.579E+03 –	6.548E+03 9.790E+02 –	4.15E+03 6.90E+02 –	6.88E+03 3.12E+02 –	4.11E+03 8.53E+02
F12	2.585E+00 3.545E−02 –	2.816E+00 4.494E−01 –	2.124E+00 1.109E−01 –	1.788E+00 6.116E−01 –	4.33E −01 2.64E −01 +	2.49E+00 2.73E–01 –	1.15E+00 6.70E−01
F13	7.508E−01 1.365E−01 –	5.184E−01 9.286E−02 –	5.725E−01 3.004E−02 –	6.601E−01 1.276E−01 –	2.21E+00 1.34E+00 –	1.08E+00 1.19E −01 –	5.10E−01 1.30E−01
F14	1.919E−01 2.441E−02 +	5.586E−01 5.275E−01 –	2.635E−01 4.831E−02 +	2.515E−01 1.881E−02 +	3.54E+01 2.47E+01 –	4.33E+00 1.70E+00 –	3.00E−01 5.00E−02
F15	1.084E+03 1.878E+02 –	2.292E+03 1.454E+03 –	1.119E+03 5.438E+02 –	3.462E+03 1.196E+03 –	2.23E+05 5.77E+05 –	5.05E+01 9.36E+00 –	4.55E+01 1.21E+01
F16	1.392E+01 6.834E−01 –	1.262E+01 4.312E−01 –	1.322E+01 3.863E−01 –	1.381E+01 9.966E−01 –	1.27E+01 5.33E −01 –	1.28E+01 1.78E −01 –	1.17E+01 4.00E−01
F17	1.179E+06 8.704E+05 –	1.935E+06 1.618E+06 –	3.150E+06 3.673E+06 –	1.573E+06 7.309E+05 –	3.39E+06 4.07E+06 –	2.63E+06 9.76E+05 –	7.82E+05 3.34E+05
F18	6.615E+03 4.575E+03 –	8.758E+03 1.307E+04 –	2.412E+03 2.713E+03 +	4.646E+03 5.907E+03 –	5.19E+06 3.61E+07 –	1.26E+07 1.06E+07 –	4.34E+03 4.79E+03
F19	1.944E+02 3.741E+01 –	3.112E+02 1.479E+02 –	1.433E+02 1.725E+01 –	1.533E+02 6.006E+01 –	7.36E+01 5.32E+01 –	3.78E+01 3.45E+01 –	2.39E+01 2.67E+01
F20	1.214E+04 5.535E+03 –	9.243E+03 7.954E+03 –	3.171E+04 3.588E+04 –	1.631E+04 1.192E+04 –	5.67E+04 4.34E+04 –	9.92E+03 3.69E+03 –	7.76E+03 4.60E+03
F21	6.694E+05 1.892E+05 –	9.029E+05 5.565E+05 –	2.959E+05 1.836E+05 +	1.346E+06 3.493E+05 –	7.83E+05 1.18E+06 –	6.94E+05 2.03E+05 –	4.87E+05 2.88E+05
F22	9.721E+02 1.592E+02 +	1.358E+03 4.080E+02 –	1.276E+03 2.694E+02 –	1.064E+03 1.124E+02 –	8.67E+02 2.29E+02 +	5.47E+02 1.05E+02 +	9.87E+02 1.88E+02
F23	2.801E+02 6.936E+01 +	3.231E+02 4.920E+00 –	3.208E+02 2.654E+00 –	3.194E+02 7.377E−01 –	3.71E+02 3.98E+01 –	3.43E+02 3.41E+00 –	3.15E+02 2.00E−02
F24	2.094E+02 5.532E+00 +	2.052E+02 3.837E+00 +	2.115E+02 2.001E+00 +	2.065E+02 3.504E+00 +	2.76E+02 2.73E+01 –	2.57E+02 4.04E+00 –	2.18E+02 1.12E+01
F25	2.406E+02 3.567E+01 –	2.293E+02 2.535E+01 –	2.187E+02 1.509E+01 +	2.149E+02 2.588E+01 +	2.14E+02 7.65E+00 +	2.16E+02 2.58E+00 +	2.23E+02 1.63E+01
F26	1.625E+02 2.418E−01 –	1.775E+02 7.831E−02 –	1.669E+02 5.742E+01 –	3.383E+02 1.202E+02 –	1.03E+02 1.50E+00 +	1.01E+02 1.02E −01 +	1.15E+02 3.76E+01
F27	1.259E+03 5.684E+02 –	1.434E+03 1.252E+02 –	1.423E+03 7.397E+01 –	1.501E+03 1.540E+01 –	9.21E+02 2.23E+02 +	9.86E+02 2.48E+02 +	1.11E+03 8.11E+01
F28	3.454E+03 5.202E+02 –	3.811E+03 5.758E+02 –	3.228E+03 9.435E+02 –	2.205E+03 1.751E+03 –	1.12E+03 1.57E+02 +	1.13E+03 6.63E+01 +	1.88E+03 8.69E+02
F29	7.767E+07 9.097E+07 –	8.864E+07 7.061E+07 –	1.572E+08 1.001E+08 –	1.117E+08 7.572E+07 –	3.06E+06 3.62E+06 +	9.82E+05 2.07E+06 +	7.54E+06 5.27E+06
F30	8.012E+04 3.813E+04 –	9.153E+04 8.882E+04 –	8.800E+04 1.611E+04 –	3.903E+05 5.016E+05 –	5.89E+04 5.40E+04 –	1.09E+04 4.24E+03 –	3.91E+03 1.91E+03
Nw/Nb/Ne	24/4/0	29/1/0	25/5/0	26/4/0	22/8/0	24/6/0	

In this table, the symbols ‘=’, ‘−’, and ‘+’ are used to indicate the comparison between the method under consideration and the proposed PDWOA. The symbol ‘=’ represents an equal result, ‘−’ indicates that the method performs worse than the proposed PDWOA, and ‘+’ signifies that the method performs better than the proposed PDWOA. Furthermore, Nw, Nb, and Ne represent the number of times the considered method performs worse than, better than, or equal to the proposed PDWOA, respectively. The table presents a comprehensive comparison of the results achieved by PDWOA in relation to the benchmarked algorithms, shedding light on the efficacy and competitiveness of PDWOA in addressing the CEC 2014 test functions.

### Statistical analysis

In this subsection, we present the results of two non-parametric statistical tests conducted to assess the performance of the proposed improved versions of the whale optimization algorithm (WOA), namely “PDWOA/Cr = rand”, “PDWOA/Cr = 0.1’, and “PDWOA/Cr = 0.9’. The tests include the Wilcoxon signed-rank test and the Friedman test, which provide insights into the algorithm rankings and pairwise comparisons.

The Friedman test was used to rank the algorithms based on their mean performance across all benchmark functions. The results of the Friedman test are presented in Table 4. In this table, the mean rank index represents the average of the Rank indices of each algorithm for all test functions, while the RankT index shows the rank of each algorithm in the list of sorted mean rank indices. Specifically, PDWOA/Cr = rand achieved the best performance with the lowest mean rank of 3.0167, followed by PDWOA/Cr = 0.1 (mean rank: 3.9), PSO (mean rank: 5.9), and PDWOA/Cr = 0.9 (mean rank: 5.9667).

Additionally, the results of the Wilcoxon signed-rank test with a significance level of 0.05 are presented in Table 5, showing the *p*-values and confidence intervals for pairwise comparisons between PDWOA/Cr = rand and other algorithms. In this table, SoPR and SoNR represent the combined positive and negative ranks. Similarly, MoPR and MoNR represent the average positive and negative ranks, respectively. The notation F(i)<F(j) indicates how many times the first algorithm performs better than the second one, while F(j)<F(i) signifies the opposite scenario. It’s important to highlight that in the Wilcoxon test, positive ranks correspond to cases where the first algorithm surpasses the second one. The test results reveal statistically significant differences in performance between PDWOA/Cr = rand and several other algorithms.

**Table 4:** Average ranking of different algorithms according to the Friedman test.

Algorithm	RankT	Mean rank
PDWOA/*Cr*= rand	1	3.0167
PDWOA/*Cr*= 0.1	2	3.9
PSO	3	5.9
PDWOA/*Cr*= 0.9	4	5.9667
WOA	5	6.1333
MFO	6	7
LJA	7	7.4333
AOA	8	7.4667
AWPSO	9	7.5333
GOMGBO	10	7.7667
DE/best/1	11	7.8833
HMCTLBO	12	8

**Table 5:** The Wilcoxon signed-rank test results between PDWOA/Cr=rand and other algorithms.

i	j	MoPR	MoNR	SoPR	SoNR	F(i) <F(j)	F(j) <F(i)	*p*-value	0.95 Confidence interval	
PDWOA/Cr = rand	DE/rand/1	16.261	13	374	91	23	7	2.766e−03	−126499.9	−40.05
	PSO	15.52	15.4	388	77	25	5	8.718e−04	-23442	−22.7
	WOA	16.087	10.833	370	65	23	6	1.014e−03	−47945.13	−24.0
	PDWOA/Cr = 0.1	15.15	14.667	303	132	20	9	6.607e−02	−2503.0	5.3
	PDWOA/Cr = 0.9	14.88	15.75	372	63	25	4	8.685e−04	−3895.5	−36.05
	AOA	16.808	7	437	28	26	4	2.762e−06	−91195.7	−86.36
	HMCTLBO	15.69	10	455	10	29	1	8.009e−08	−207950.13	−193.25
	AWPSO	16.04	12.8	401	64	25	5	2.563e−04	−42041.75	−69.45
	GOMGBO	16.808	7	437	28	26	4	2.762e−06	−395495.95	−153.21
	MFO	16.682	12.25	367	98	22	8	4.665e−03	-147940	−25.35
	LJA	15.375	16	369	96	24	6	4.032e−03	−103125	−8.97

PDWOA/Cr = rand was found to have significantly better performance compared to DE/rand/1, PSO, WOA, PDWOA/Cr = 0.9, AOA, AWPSO, GOMGBO, MFO, and LJA, as indicated by the low *p*-values obtained. The confidence intervals further support this finding, showing that PDWOA/Cr = rand consistently outperformed these algorithms over a wide range of objective function values.

However, when comparing PDWOA/Cr = rand with PDWOA/Cr = 0.1, the obtained *p*-value (0.0661) suggests that the difference in performance between these two algorithms is not statistically significant at the 0.05 significance level. It is worth noting that PDWOA/Cr = rand still exhibits a slightly better performance trend.

Overall, the results of the statistical tests support the superiority of PDWOA/Cr = rand compared to the other algorithms tested. It demonstrates consistent and competitive performance, as evidenced by its lower mean rank in the Friedman test and its significant performance advantages in the pairwise comparisons based on the Wilcoxon signed-rank test.

### PDWOA for solving constrained engineering optimization

So as to further demonstrate the optimization power of the suggested algorithm, we have selected three renowned engineering problems and solved them with the proposed method. To solve these problems, the population sizes selected for each algorithm is 60 and the number of iterations of each algorithm for each run is 1,000. For each problem, optimization was performed in 30 independent runs. All parameters of the algorithms used here are exactly according to the main references suggested by the algorithm designers.

#### Pressure vessel optimal design (engineering problem 1)

**Figure 4:**
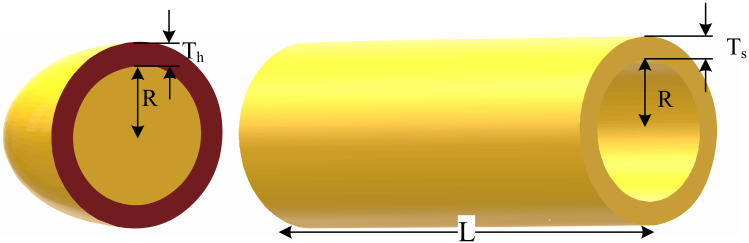
Schematic of the pressure vessel design problem.

The problem is focused on optimally finding two discrete (*x*_1_ and *x*_2_) and two continuous (*x*_3_ and *x*_4_) decision variables for the minimization of the cost of a pressure vessel (Fig. 4) subject to three linear and one nonlinear inequality constraint. The optimization variables are the thickness of the shell (*x*_1_ or *T*_*s*_), the thickness of the head (*x*_2_ or *T*_*h*_), the inner radius (*x*_3_ or *R*), and the length of the cylindrical part of the vessel (*x*_4_ or *L*) (Askarzadeh, 2016).

Minimize:

(18) \begin{eqnarray*}f \left( X \right) =0.6224{x}_{1}{x}_{3}{x}_{4}+1.7781{x}_{2}{x}_{3}^{2}+3.1661{x}_{1}^{2}{x}_{4}+19.84{x}_{1}^{2}{x}_{3}\end{eqnarray*}

subject to:

(19) \begin{eqnarray*}{g}_{1} \left( X \right) =-{x}_{1}+0.0193{x}_{3}\leq 0,\end{eqnarray*}

(20) \begin{eqnarray*}{g}_{2} \left( X \right) =-{x}_{2}+0.00954{x}_{3}\leq 0,\end{eqnarray*}

(21) \begin{eqnarray*}{g}_{3} \left( X \right) =-\pi {x}_{3}^{2}{x}_{4}- \frac{4}{3} \pi {x}_{3}^{3}+1,296,000\leq 0,\end{eqnarray*}

(22) \begin{eqnarray*}{g}_{4} \left( X \right) ={x}_{4}-240\leq 0,\end{eqnarray*}

where *x*_1_, *x*_2_ ∈ [0, 100], and *x*_3_, *x*_4_ ∈ [10, 200].

Table 6 presents the results of PDWOA in solving this problem compared to several new algorithms, including quantum-behaved PSO (QPSO) (Coelho L dos, dos Santos Coelho & Coelho L dos, 2010), ABC (Akay & Karaboga, 2012), GA enhanced with dominance-based tournament selection (GA4) (Coello Coello et al., 2002), co-evolutionary PSO (CPSO) (He & Wang, 2007), co-evolutionary DE (CDE) (Huang, Wang & He, 2007), gaussian quantum-behaved PSO (G-QPSO) (Coelho L dos, dos Santos Coelho & Coelho L dos, 2010), Unified PSO (UPSO) (Parsopoulos & Vrahatis, 2005), Crow search algorithm (CSA) (Askarzadeh, 2016), hybrid GA and artificial immune system (HAIS-GA) (Coello & Cortés, 2004), bacterial foraging optimization algorithm (BFOA) (Mezura-Montes & Hernández-Ocana, 2008), evolution strategies (ES) (Mezura-Montes & Coello, 2008), modified T-Cell Algorithm (Aragón, Esquivel & Coello, 2010), GA enhanced with self-adaptive penalty approach (GA3) (Coello Coello, 2000), Queuing search (QS) algorithm (Zhang et al., 2018), and automatic dynamic penalization (ADP) for GA (BIANCA) (Montemurro, Vincenti & Vannucci, 2013), K-means optimizer (KO) (Minh et al., 2022), and termite life cycle optimizer (TLCO) (Minh et al., 2023b; Minh et al., 2023a). The best solutions for the considered problem found using the original and proposed versions of WOA are presented in Table 7. The results demonstrate the effectiveness of the proposed PDWOA in achieving high-quality solutions for the optimization problem.

**Table 6:** Best statistical results of various algorithms for engineering problem 1.

Methods	Best	Mean	Worst	Std.
QPSO (Coelho L dos, dos Santos Coelho & Coelho L dos, 2010)	6059.7209	6440.3786	8017.2816	479.2671
ABC (Akay & Karaboga, 2012)	6059.714339	6245.308144	N.A.	2.05e+02
GA4 (Coello Coello et al., 2002)	6059.9463	6177.2533	6469.3220	130.9297
CPSO (He & Wang, 2007)	6061.0777	6147.1332	6363.8041	86.4545
CDE (Huang, Wang & He, 2007)	6059.7340	6085.2303	6371.0455	43.013
G-QPSO (Coelho L dos, dos Santos Coelho & Coelho L dos, 2010)	6059.7208	6440.3786	7544.4925	448.4711
UPSO (Parsopoulos & Vrahatis, 2005)	6154.70	8016.37	9387.77	745.869
ES (Mezura-Montes & Coello, 2008)	6059.746	6850.00	7332.87	426
T-Cell (Aragón, Esquivel & Coello, 2010)	6390.554	6737.065	7694.066	357
GA3 (Coello Coello, 2000)	6288.7445	6293.8432	6308.4970	7.4133
HAIS-GA (Coello & Cortés, 2004)	6832.584	7187.314	8012.615	276
CSA (Askarzadeh, 2016)	6059.71436343	6342.49910551	7332.84162110	384.94541634
BFOA (Mezura-Montes & Hernández-Ocana, 2008)	6060.460	6074.625	N.A.	156
BIANCA (Montemurro, Vincenti & Vannucci, 2013)	6059.9384	6182.0022	6447.3251	122.3256
QS (Zhang et al., 2018)	6059.714	6060.947	6090.526	N.A.
KO	6059.71475731827	6059.72453197228	N.A.	0.005942
TLCO	6059.71433504844	N.A.	N.A.	N.A.
WOA	6059. 823537	6115.250471	6314.025148	62.35
PDWOA/*Cr*= 0.9	6059.714335	6064.922632	6084.003815	12.94
PDWOA/*Cr*= rand	6059.714335	6063.175326	6090.742650	24.65
PDWOA/*Cr*= 0.1	6059.714335	6060.789025	6064.324186	1.83

**Table 7:** The best solutions for engineering problem 1.

Design variables	WOA	PDWOA/*Cr*= 0.9	PDWOA/*Cr*= rand	PDWOA/*Cr*= 0.1
*x*_1_	0.8125	0.8125	0.8125	0.8125
*x*_2_	0.4375	0.4375	0.4375	0.4375
*x*_3_	42.09765834	42.09844559	42.09844559	42.09844559
x_4_	176.6469213	176.63659592	176.63659592	176.63659592
*g*_1_(*X*)	−1.519403799987718e−05	−1.130000537585829e−10	−1.130000537585829e−10	−1.130000537585829e−10
*g*_2_(*X*)	−0.0358883394364	−0.035880829071400	−0.035880829071400	−0.035880829071400
*g*_3_(*X*)	−3.172713808366098	−2.788752317428589e−05	−2.788752317428589e−05	−2.788752317428589e−05
*g*_4_(*X*)	−63.353078699999998	−63.363404080000009	−63.363404080000009	−63.363404080000009
Best	6059. 823537	6059.714335	6059.714335	6059.714335

#### Tension/compression spring optimal design (engineering problem 2)

**Figure 5:**
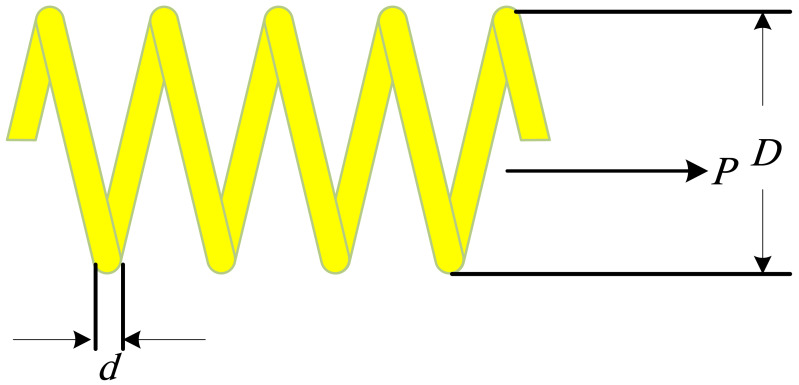
The tension/compression spring optimal design problem.

The problem involves finding three continuous decision variables to minimize the weight of the spring (Fig. 5). The optimization variables are the wire diameter (*d* or *x*_1_), mean coil diameter (*D* or *x*_2_), and the number of active coils (*P* or *x*_3_), subject to one linear and three nonlinear inequality constraints (Askarzadeh, 2016).

Minimize:

(23) \begin{eqnarray*}f \left( X \right) = \left( {x}_{3}+2 \right) {x}_{2}{x}_{1}^{2}\end{eqnarray*}

subject to:

(24) \begin{eqnarray*}{g}_{1} \left( X \right) =1- \frac{{x}_{2}^{3}{x}_{3}}{71,785{x}_{1}^{4}} \leq 0,\end{eqnarray*}

(25) \begin{eqnarray*}{g}_{2} \left( X \right) = \frac{4{x}_{2}^{2}-{x}_{1}{x}_{2}}{12,566 \left( {x}_{1}^{3}{x}_{2}-{x}_{1}^{4} \right) } + \frac{1}{5,108{x}_{1}^{2}} -1\leq 0,{x}_{3}\in [2,15]\end{eqnarray*}

(26) \begin{eqnarray*}{g}_{3} \left( X \right) =1- \frac{140.45{x}_{1}}{{x}_{2}^{2}{x}_{3}} \leq 0,\end{eqnarray*}

(27) \begin{eqnarray*}{g}_{4} \left( X \right) = \frac{{x}_{1}+{x}_{2}}{1.5} -1\leq 0.\end{eqnarray*}

In which *x*_1_ ∈ [0.05, 2], *x*_2_ ∈ [0.25, 1.3], and *x*_2_ ∈ [2, 15].

Table 8 compares the results of the proposed method for solving the engineering problem 2 with several other algorithms, including BFOA (Mezura-Montes & Hernández-Ocana, 2008), T-Cell (Aragón, Esquivel & Coello, 2010), CDE (Huang, Wang & He, 2007), CPSO (He & Wang, 2007), a cultural algorithm (CA) (Coello Coello & Becerra, 2004), GA4 (Coello Coello et al., 2002), GA3 (Coello Coello, 2000), TEO (Kaveh & Dadras, 2017), G-QPSO (Coelho L dos, dos Santos Coelho & Coelho L dos, 2010), SBO (Ray & Liew, 2003), evolutionary algorithms ((l + k)-ES) (Mezura-Montes & Coello, 2005), UPSO (Parsopoulos & Vrahatis, 2005), Grey wolf optimizer (GWO) (Mirjalili, Mirjalili & Lewis, 2014), SDO (Zhao, Wang & Zhang, 2019), QS (Zhang et al., 2018), Water cycle algorithm (WCA) (Eskandar et al., 2012), BIANCA (Montemurro, Vincenti & Vannucci, 2013), KO (Minh et al., 2022), TLCO (Minh et al., 2023b; Minh et al., 2023a), planet optimization algorithm (POA) (Sang-To et al., 2022), Cuckoo Search Algorithm (CS) (Cuong-Le et al., 2021), and the new movement strategy of cuckoo search (NMS-CS) (Cuong-Le et al., 2021). Table 9 provides the best solutions obtained by the original and proposed versions of WOA for this problem. The findings indicate that the suggested PDWOA is successful in attaining excellent solutions for the optimization issue.

**Table 8:** Best statistical results of various algorithms for engineering problem 2.

Methods	Best	Mean	Worst	Std.
CA (Coello Coello & Becerra, 2004)	0.012721	0.013568	0.0151156	8.4e−04
BFOA (Mezura-Montes & Hernández-Ocana, 2008)	0.012671	0.012759	N.A.	1.36e−04
T-Cell (Aragón, Esquivel & Coello, 2010)	0.012665	0.012732	0.013309	9.4e−05
CDE (Huang, Wang & He, 2007)	0.012670	0.012703	0.012790	2.07e−05
CPSO (He & Wang, 2007)	0.0126747	0.012730	0.012924	5.19e−05
TEO (Kaveh & Dadras, 2017)	0.012665	0.012685	0.012715	4.4079e−06
G-QPSO (Coelho L dos, dos Santos Coelho & Coelho L dos, 2010)	0.012665	0.013524	0.017759	1.268e−03
SBO (Ray & Liew, 2003)	0.012669249	0.012922669	0.016717272	5.92e−04
GA4 (Coello Coello et al., 2002)	0.012681	0.012742	0.012973	9.5e−05
GA3 (Coello Coello, 2000)	0.0127048	0.012769	0.012822	3.93e−05
(l + k)-ES (Mezura-Montes & Coello, 2005)	0.012689	0.013165	N.A.	3.9e−04
UPSO (Parsopoulos & Vrahatis, 2005)	0.01312	0.02294	N.A.	7.2e−03
GWO (Mirjalili, Mirjalili & Lewis, 2014)	0.0126660	N.A.	N.A.	N.A.
WCA (Eskandar et al., 2012)	0.012665	0.012746	0.012952	8.06e−05
BIANCA (Montemurro, Vincenti & Vannucci, 2013)	0.012671	0.012681	0.012913	5.1232e−05
SDO (Zhao, Wang & Zhang, 2019)	0.0126663	0.0126724	0.0126828	6.1899e−06
QS (Zhang et al., 2018)	0.012665	0.012666	0.012669	N.A.
KO	0.012665994	0.012917292	N.A.	0.00030139
TLCO	0.0126652328	N.A.	N.A.	N.A.
POA	0.01266588	N.A.	N.A.	N.A.
CS	0.012665871	N.A.	N.A.	N.A.
NMS-CS	0.012665233	N.A.	N.A.	N.A.
WOA	0.012667	0.013586	0.018416	5.05e−03
PDWOA/*Cr*= 0.9	0.012665	0.012695	0.012842	8.75e−06
PDWOA/*Cr*= rand	0.012665	0.012706	0.012907	1.18e−05
PDWOA/*Cr*= 0.1	0.012665	0.012665	0.012666	9.22e−08

**Table 9:** The best solutions for engineering problem 2.

Design variables	WOA	PDWOA/*Cr*= 0.9	PDWOA/*Cr*= rand	PDWOA/*Cr*= 0.1
*x*_1_	0.0517315934	0.0515902788	0.0516488707	0.0516911532
*x*_2_	0.3577231396	0.3543444741	0.3557507777	0.3567674033
*x*_3_	11.2318481822	11.4295493851	11.3460406066	11.2862994555
*g*_1_(*X*)	−8.105263015556474e−05	−2.067013876949631e−06	−1.124305991995200e−05	−1.953083625561014e−05
*g*_2_(*X*)	−4.202663105634663e−05	−3.336199576375876e−06	−1.936754291387288e−06	−1.509602815197297e−06
*g*_3_(*X*)	−4.055129747949155	−4.049044297898749	−4.051804364878148	−4.053776839282882
*g*_4_(*X*)	−0.727030178	−0.729376831400	−0.7284002344	−0.727694295666667
Best	0.012667	0.012665	0.012665	0.012665

#### Welded beam optimal design (engineering problem 3)

**Figure 6:**
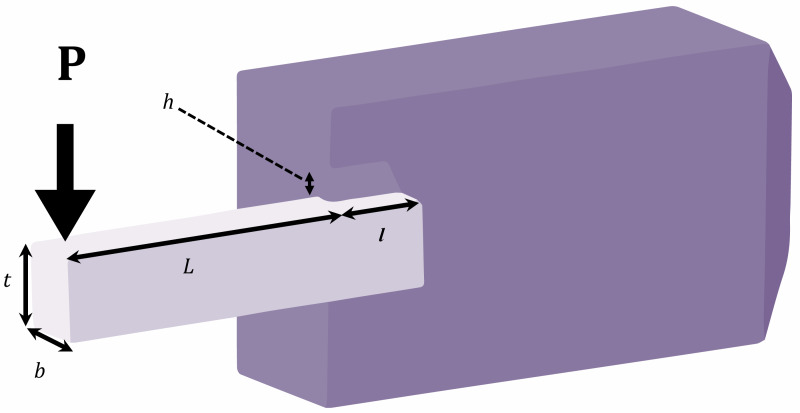
Schematic of welded beam optimal design problem.

The problem is focused on optimally finding four continuous decision variables for minimizing the cost of a welded beam (Fig. 6) subject to two linear and five nonlinear inequality constraints. The optimization variables are *x*_1_ or h, *x*_2_ or *l*, *x*_3_ or *t*, and *x*_4_ or *b* (Askarzadeh, 2016).

Minimize:

(28) \begin{eqnarray*}f \left( X \right) =1.10471{x}_{2}{x}_{1}^{2}+0.04811{x}_{3}{x}_{4} \left( 14+{x}_{2} \right) \end{eqnarray*}

subject to:

(29) \begin{eqnarray*}{g}_{1} \left( X \right) =\tau \left( x \right) -{\tau }_{\mathrm{max}}\leq 0,\end{eqnarray*}

(30) \begin{eqnarray*}{g}_{2} \left( X \right) =\sigma \left( x \right) -{\sigma }_{\mathrm{max}}\leq 0,\end{eqnarray*}

(31) \begin{eqnarray*}{g}_{3} \left( X \right) ={x}_{1}-{x}_{4}\leq 0,\end{eqnarray*}

(32) \begin{eqnarray*}{g}_{4} \left( X \right) =0.10471{x}_{1}^{2}+0.04811{x}_{3}{x}_{4} \left( 14+{x}_{2} \right) -5\leq 0.\end{eqnarray*}

(33) \begin{eqnarray*}{g}_{5} \left( X \right) =0.125-{x}_{1}\leq 0,\end{eqnarray*}

(34) \begin{eqnarray*}{g}_{6} \left( X \right) =\delta \left( x \right) -{\delta }_{\mathrm{max}}\leq 0,\end{eqnarray*}

(35) \begin{eqnarray*}{g}_{7} \left( X \right) =P-{P}_{c} \left( x \right) \leq 0,\end{eqnarray*}

(36) \begin{eqnarray*}\tau \left( x \right) =\sqrt{{ \left( {\tau }^{{}^{{^{\prime}}}} \right) }^{2}+2{\tau }^{{}^{{^{\prime}}}}{\tau }^{{^{\prime}}{^{\prime}}} \frac{{x}_{2}}{2R} +{ \left( {\tau }^{{^{\prime}}{^{\prime}}} \right) }^{2}}\end{eqnarray*}

(37-38) \begin{eqnarray*}{\tau }^{{}^{{^{\prime}}}}= \frac{P}{\sqrt{2}{x}_{1}{x}_{2}} ,\,{\tau }^{{^{\prime}}{^{\prime}}}= \frac{MR}{J} ,\end{eqnarray*}

(39-41) \begin{eqnarray*}M=P \left( L+ \frac{{x}_{2}}{2} \right) ,\,R=\sqrt{ \frac{{x}_{2}^{2}}{4} +{ \left( \frac{{x}_{1}+{x}_{3}}{2} \right) }^{2}},\,\,\delta \left( x \right) = \frac{4P{L}^{3}}{E{x}_{3}^{3}{x}_{4}} \end{eqnarray*}

(42-43) \begin{eqnarray*}J=2 \left[ \sqrt{2}{x}_{1}{x}_{2} \left\{ \frac{{x}_{2}^{2}}{12} +{ \left( \frac{{x}_{1}+{x}_{3}}{2} \right) }^{2} \right\} \right] ,\,\sigma \left( x \right) = \frac{6PL}{{x}_{4}{x}_{3}^{2}} ,\end{eqnarray*}

(44) \begin{eqnarray*}{P}_{c} \left( x \right) = \frac{4.013E\sqrt{ \frac{{x}_{4}^{6}{x}_{3}^{2}}{36} }}{{L}^{2}} \left( 1- \frac{{x}_{3}}{2L} \sqrt{ \frac{E}{4G} } \right) ,\,\,\end{eqnarray*}

where *P* = 6,000 lb; *L* = 14 in; *E* = 30e6 psi; *G* = 12e6 psi; *τ*_max_ =13,000 psi; *σ*_max_ =30,000 psi; *δ*_max_ = 0.25 in; *x*_1_ ∈ [0.1, 2]; *x*_2_ ∈ [0.1, 10]; *x*_3_ ∈ [0.1, 10]; and *x*_4_ ∈ [0.1, 2].

Table 10 presents the results of the proposed method for solving the engineering problem 3 in comparison to several other algorithms, including a cooperative PSO with stochastic movements (EPSO) (Ngo, Sadollah & Kim, 2016), BFOA (Mezura-Montes & Hernández-Ocana, 2008), T-Cell Algorithm (Aragón, Esquivel & Coello, 2010), CDE (Huang, Wang & He, 2007), CPSO (He & Wang, 2007), Derivative-Free Filter Simulated Annealing Method (FSA) (Hedar & Fukushima, 2006), TEO (Kaveh & Dadras, 2017), SBO (Ray & Liew, 2003), GA4 (Coello Coello et al., 2002), (l + k)-ES (Mezura-Montes & Coello, 2005), UPSO (Parsopoulos & Vrahatis, 2005), GWO (Mirjalili, Mirjalili & Lewis, 2014), SFO (Shadravan, Naji & Bardsiri, 2019), HGSO (Hashim et al., 2019), WCA (Eskandar et al., 2012), BIANCA (Montemurro, Vincenti & Vannucci, 2013), SBO (Ray & Liew, 2003), KO (Minh et al., 2022), TLCO (Minh et al., 2023b; Minh et al., 2023a), POA (Sang-To et al., 2022), CS (Cuong-Le et al., 2021), and NMS-CS (Cuong-Le et al., 2021). Table 11 presents the best solutions found by the original and proposed versions of WOA for this problem. The outcomes exhibit the efficacy of the suggested PDWOA in attaining top-notch solutions for the optimization issue.

**Table 10:** Best statistical results of various algorithms for engineering problem 3.

Methods	Best	Mean	Worst	Std.
EPSO (Ngo, Sadollah & Kim, 2016)	1.7248530	1.7282190	1.7472200	5.62e−03
BFOA (Mezura-Montes & Hernández-Ocana, 2008)	2.3868	2.4040	N.A.	1.6e−02
T-Cell (Aragón, Esquivel & Coello, 2010)	2.3811	2.4398	2.7104	9.314e−02
CDE (Huang, Wang & He, 2007)	1.73346	1.768158	1.824105	2.2194e−02
CPSO (He & Wang, 2007)	1.728024	1.748831	1.782143	1.2926e−02
FSA (Hedar & Fukushima, 2006)	2.3811	2.4041	2.4889	N.A.
TEO (Kaveh & Dadras, 2017)	1.725284	1.768040	1.931161	5.81661e−02
SBO (Ray & Liew, 2003)	2.3854347	3.0025883	6.3996785	9.59e−01
GA4 (Coello Coello et al., 2002)	1.728226	1.792654	1.993408	7.47e−02
(l + k)-ES (Mezura-Montes & Coello, 2005)	1.724852	1.777692	N.A.	8.8e−02
UPSO (Parsopoulos & Vrahatis, 2005)	1.92199	2.83721	N.A.	6.83e−01
GWO (Mirjalili, Mirjalili & Lewis, 2014)	1.72624	N.A.	N.A.	N.A.
SFO (Shadravan, Naji & Bardsiri, 2019)	1.73231	N.A.	N.A.	N.A.
HGSO (Hashim et al., 2019)	1.7260	1.7265	1.7325	7.66e−03
WCA (Eskandar et al., 2012)	1.724856	1.726427	1.744697	4.29e−03
BIANCA (Montemurro, Vincenti & Vannucci, 2013)	1.725436	1.752201	1.793233	2.3001e−02
KO	1.725344872	1.75727933	N.A.	0.029125
TLCO	1.724852433	N.A.	N.A.	N.A.
POA	1.72564	N.A.	N.A.	N.A.
CS	1.73139841	N.A.	N.A.	N.A.
NMS-CS	1.72620872	N.A.	N.A.	N.A.
WOA	1.7273929	2.2852435	3.2784166	2.62
PDWOA/*Cr*= 0.9	1.7248523	1.7310629	1.7491305	9.83e−04
PDWOA/*Cr*= rand	1.7248523	1.7588375	1.7709064	2.06e−03
PDWOA/*Cr*= 0.1	1.7248523	1.7259521	1.7340485	5.93e−05

**Table 11:** The best solutions for engineering problem 3.

Design variables	WOA	PDWOA/*Cr*= 0.9	PDWOA/*Cr*= rand	PDWOA/*Cr*= 0.1
*x*_1_	0.2053718352	0.2057296398	0.20572963980	0.20572963980
*x*_2_	3.4771582193	3.470488670	3.4704886655	3.4704886655
*x*_3_	9.0472014495	9.0366239108	9.0366239101	9.0366239101
x_4_	0.2057779509	0.2057296398	0.2057296398	0.2057296398
*g*_1_(*X*)	−9.453362733127506	−1.514258474344388e−05	−2.265333023387939e−07	−2.265333023387939e−07
*g*_2_(*X*)	−77.134747837790201	−4.967081622453407e−06	−3.193272277712822e−07	−3.193272277712822e−07
*g*_3_(*X*)	−4.061157000000149e−04	0.0	0.0	0.0
*g*_4_(*X*)	−3.43020541215535	−3.432983784788387	−3.432983785311915	−3.432983785311915
g_5_(X)	−0.08037183520	−0.08072963980	−0.080729639800	−0.080729639800
g_6_(X)	−0.235594362774535	−0.235540322587856	−0.235540322584496	−0.235540322584496
g_7_(X)	−8.845720840467948	−1.411061930411961e−06	−1.105492628994398e−06	−1.105492628994398e−06
Best	1.7273929	1.7248523	1.7248523	1.7248523

## Discussion and Future Studies

Table 12 presents the best solutions found by the proposed and original versions of WOA. The results indicate that optimizing the algorithm’s parameters, particularly the value of *Cr*, can significantly enhance the optimization performance. For example, when comparing the final results for F29 (shown in Table 1), the original WOA yielded the best mean value with a slight difference, whereas the proposed algorithm with *Cr* equal to 0.1 achieved the best final value, as demonstrated in Table 12. As part of future work, an efficient modification can be explored to improve the Mean obtained by PDWOA and align it with the best value.

**Table 12:** Summary of the best results for CEC 2014 test functions for WOA algorithms.

Function	WOA	PDWOA/ *Cr*= rand	PDWOA/ *Cr* = 0.1	PDWOA/ *Cr* = 0.9
	Best	Best	Best	Best
F1	19833281.23	680089.84	1348332.9	1288387.42
F2	1994653.36	1105.87	1.24	93.08
F3	15105.52	101.54	48.61	1950.79
F4	132.74	67.85	78.74	73.05
F5	20.08	20	20	19.99
F6	31.78	28.38	21.54	37.93
F7	0.97	1.94e−5	2.55e−6	1.28e−3
F8	137.4	156.99	38.8	128.35
F9	176.36	143.27	138.3	190.04
F10	2543.45	2428.22	44.13	3793.69
F11	3809.73	3456.96	2876.84	2874.24
F12	0.43	0.69	0.18	1.17
F13	0.4	0.45	0.39	0.44
F14	0.21	0.23	0.22	0.25
F15	50.75	191.85	22.36	375.04
F16	11.81	12.65	11.14	12.12
F17	2514752.92	211439.89	477893.91	420326.09
F18	483.1	270.89	92.49	215.83
F19	24.86	17.97	10.33	36.43
F20	8808.02	350.79	635.06	958.96
F21	290044.26	126426.49	213375.89	44587.71
F22	451.24	446.64	580.79	896.22
F23	324.86	315.26	315.25	315.3
F24	201.88	201.38	202.81	202.16
F25	200	200	205.68	200
F26	100.35	100.57	100.23	100.49
F27	459.35	409.61	1015.92	444.06
F28	1576.31	200	200	200
F29	38445.29	1364.48	1141.58	1123.79
F30	52816	5011.8	2201.07	5668.48

**Figure 7:**
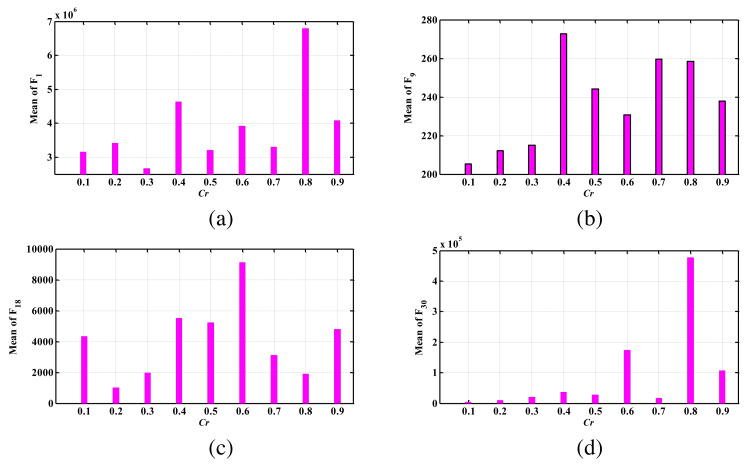
The mean index obtained for different test functions by PDWOA having different Cr values; (A) F1 (unimodal); (B) F9 (simple multimodal); (C) F18 (hybrid); (D) F30 (composition).

Figure 7 illustrates the average performance of the suggested algorithm across multiple test functions in 30 runs. These results demonstrate the significant improvement achieved by the suggested algorithm in enhancing WOA. Although the suggested algorithm is robust and effective in many cases, further development can be pursued by exploring numerous enhanced versions of PSO or DE. In future work, we will present some examples of these enhanced versions. For instance, we can draw inspiration from the colonial competitive differential evolution algorithm (Ghasemi et al., 2016), which suggests distributing the population into several groups and conducting colonial competition between these groups using a specific mutation for each group. To enhance the proposed version, we can implement a similar mechanism by dividing the WOA population into multiple groups, where the best member of each group serves as the leader, and conduct colonial competition among these groups.

Additionally, instead of using the specific mutation equation defined in Eq. (17), we can employ alternative mutations (or crossover coefficients) for each group of whales. By leveraging the efficient operators from various evolutionary algorithms, we can increase the population diversity during iterations. This approach, guided by multiple leaders within distinct groups, allows the population to explore several different areas in the search space, effectively avoiding local optima. In this context, several new optimization algorithms that involve population division into multiple groups (Mallipeddi et al., 2011; Zhang, 2015; Chen et al., 2018; Band et al., 2022) can be applied to enhance the proposed version of WOA.

Furthermore, a highly effective and adaptive method for selecting *Cr* was proposed in Zhang & Sanderson (2009), which is recognized as one of the most powerful versions of DEs. The mutation equation presented in Eq. (17) has drawn inspiration from this method. In future studies, we can explore the application of this strategy to enhance the performance of the suggested method. Additionally, other efficient adaptive techniques proposed in Brest et al. (2006) and Zhu et al. (2013) can be further investigated as potential avenues to improve the proposed algorithm. Additionally, there are several new models of DE proposed in the literature that extend beyond the scope of this study but warrant further investigation. These models include fuzzy adaptive differential evolution (Al-Dabbagh et al., 2014), Gaussian bare-bones differential evolution (Wang et al., 2013), and parallel DE with self-adapting control parameters and generalized opposition-based learning (Wang, Rahnamayan & Wu, 2013). Future studies can delve into these models in more detail to explore their potential implications.

## Conclusions

In this study, we addressed the limitations of the original WOA, such as susceptibility to getting trapped in locally optimal solutions, particularly in complex real-world problems. To overcome these drawbacks, we proposed a new and high-performing version of WOA called PDWOA. The performance of PDWOA was evaluated by comparing it with the original WOA on 30 shifted test functions from CEC2014, each with a dimension of 30, under identical conditions. The simulation results demonstrated the efficiency of the suggested algorithm in achieving optimal solutions for the test cases. Moreover, the proposed PDWOA algorithm was evaluated using two non-parametric statistical tests, including the Friedman test and the Wilcoxon signed-rank test, which confirmed its superior performance compared to other algorithms. Furthermore, PDWOA was applied to three real-world engineering problems, providing additional evidence of its optimization performance. Additionally, we discussed models of powerful algorithms from the literature that could be explored in future studies to further enhance the proposed algorithm. By integrating these models into the proposed formulation, we aim to achieve accurate solutions for a wider range of real-world optimization problems.

## Additional Information and Declarations

### Data Availability

The following information was supplied regarding data availability: The data is available at Zenodo: Ebrahim Akbari, & Abolfazl Rahimnejad. (2023). PDWOA MATLAB Code. Zenodo. https://doi.org/10.5281/zenodo.7608100.

## References

[ref-1] Abd Elaziz M, Oliva D (2018). Parameter estimation of solar cells diode models by an improved opposition-based whale optimization algorithm. Energy Conversion and Management.

[ref-2] Abdel-Basset M, Abdle-Fatah L, Sangaiah AK (2018). An improved Lévy based whale optimization algorithm for bandwidth-efficient virtual machine placement in cloud computing environment. Cluster Computing.

[ref-3] Abdel-Basset M, Mohamed R, Mirjalili S (2021). A novel Whale optimization algorithm integrated with Nelder–Mead simplex for multi-objective optimization problems. Knowledge-Based Systems.

[ref-4] Abualigah L, Diabat A, Mirjalili S, Abd Elaziz M, Gandomi AH (2021). The arithmetic optimization algorithm. Computer Methods in Applied Mechanics and Engineering.

[ref-5] Akay B, Karaboga D (2012). Artificial bee colony algorithm for large-scale problems and engineering design optimization. Journal of Intelligent Manufacturing.

[ref-6] Akyol S, Alatas B (2020). Sentiment classification within online social media using whale optimization algorithm and social impact theory based optimization. Physica A: Statistical Mechanics and its Applications.

[ref-7] Al-Dabbagh RD, Kinsheel A, Mekhilef S, Baba MS, Shamshirband S (2014). System identification and control of robot manipulator based on fuzzy adaptive differential evolution algorithm. Advances in Engineering Software.

[ref-8] Aragón VS, Esquivel SC, Coello CAC (2010). A modified version of a T-Cell Algorithm for constrained optimization problems. International Journal for Numerical Methods in Engineering.

[ref-9] Askarzadeh A (2016). A novel metaheuristic method for solving constrained engineering optimization problems: crow search algorithm. Computers & Structures.

[ref-10] Aziz MA El, Ewees AA, Hassanien AE (2018). Multi-objective whale optimization algorithm for content-based image retrieval. Multimedia Tools and Applications.

[ref-11] Band SS, Ardabili S, Seyed Danesh A, Mansor Z, AlShourbaji I, Mosavi A (2022). Colonial competitive evolutionary Rao algorithm for optimal engineering design. Alexandria Engineering Journal.

[ref-12] Brest J, Greiner S, Boskovic B, Mernik M, Zumer V (2006). Self-adapting control parameters in differential evolution: a comparative study on numerical benchmark problems. IEEE Transactions on Evolutionary Computation.

[ref-13] Buch H, Trivedi IN, Jangir P (2017). Moth flame optimization to solve optimal power flow with non-parametric statistical evaluation validation. Cogent Engineering.

[ref-14] Canayaz M, Özdağ R (2017). Data clustering based on the whale optimization. Middle East Journal of Technic.

[ref-15] Cao Y, Li Y, Zhang G, Jermsittiparsert K, Nasseri M (2020). An efficient terminal voltage control for PEMFC based on an improved version of whale optimization algorithm. Energy Reports.

[ref-16] Chen Y, Li L, Peng H, Xiao J, Wu Q (2018). Dynamic multi-swarm differential learning particle swarm optimizer. Swarm and Evolutionary Computation.

[ref-17] Chen H, Li W, Yang X (2020). A whale optimization algorithm with chaos mechanism based on quasi-opposition for global optimization problems. Expert Systems with Applications.

[ref-18] Chen H, Yang C, Heidari AA, Zhao X (2020). An efficient double adaptive random spare reinforced whale optimization algorithm. Expert Systems with Applications.

[ref-19] Coello Coello CA (2000). Use of a self-adaptive penalty approach for engineering optimization problems. Computers in Industry.

[ref-20] Coello Coello CA, Becerra RL (2004). Efficient evolutionary optimization through the use of a cultural algorithm. Engineering Optimization.

[ref-21] Coello CAC, Cortés NC (2004). Hybridizing a genetic algorithm with an artificial immune system for global optimization. Engineering Optimization.

[ref-22] Coelho L dos S, dos Santos Coelho L, Coelho L dos S (2010). Gaussian quantum-behaved particle swarm optimization approaches for constrained engineering design problems. Expert Systems with Applications.

[ref-23] Coello Coello CA, Mezura Montes E, Coello CAC, Montes EM, Coello Coello CA, Mezura Montes E (2002). Constraint-handling in genetic algorithms through the use of dominance-based tournament selection. Advanced Engineering Informatics.

[ref-24] Cuong-Le T, Minh H-L, Khatir S, Wahab MA, Tran MT, Mirjalili S (2021). A novel version of Cuckoo search algorithm for solving optimization problems. Expert Systems with Applications.

[ref-25] Derrac J, García S, Molina D, Herrera F (2011). A practical tutorial on the use of nonparametric statistical tests as a methodology for comparing evolutionary and swarm intelligence algorithms. Swarm and Evolutionary Computation.

[ref-26] Eberhart R, Kennedy J (1995). A new optimizer using particle swarm theory.

[ref-27] Eid HF (2018). Binary whale optimisation: an effective swarm algorithm for feature selection. International Journal of Metaheuristics.

[ref-28] Eskandar H, Sadollah A, Bahreininejad A, Hamdi M (2012). Water cycle algorithm—a novel metaheuristic optimization method for solving constrained engineering optimization problems. Computers & Structures.

[ref-29] Gharehchopogh FS, Gholizadeh H (2019). A comprehensive survey: whale optimization algorithm and its applications. Swarm and Evolutionary Computation.

[ref-30] Ghasemi M, Taghizadeh M, Ghavidel S, Abbasian A (2016). Colonial competitive differential evolution: an experimental study for optimal economic load dispatch. Applied Soft Computing.

[ref-31] Ghasemi M, Zare M, Trojovský P, Zahedibialvaei A, Trojovská E (2023). A hybridizing-enhanced differential evolution for optimization. PeerJ Computer Science.

[ref-32] Guo W, Liu T, Dai F, Xu P (2020). An improved whale optimization algorithm for forecasting water resources demand. Applied Soft Computing.

[ref-33] Hashim FA, Houssein EH, Mabrouk MS, Al-Atabany W, Mirjalili S (2019). Henry gas solubility optimization: a novel physics-based algorithm. Future Generation Computer Systems.

[ref-34] He B, Huang Y, Wang D, Yan B, Dong D (2019). A parameter-adaptive stochastic resonance based on whale optimization algorithm for weak signal detection for rotating machinery. Measurement.

[ref-35] He Q, Wang L (2007). An effective co-evolutionary particle swarm optimization for constrained engineering design problems. Engineering Applications of Artificial Intelligence.

[ref-36] Hedar A-R, Fukushima M (2006). Derivative-free filter simulated annealing method for constrained continuous global optimization. Journal of Global Optimization.

[ref-37] Holland JH (1992). Genetic algorithms. Scientific American.

[ref-38] Hou G, Gong L, Yang Z, Zhang J (2020). Multi-objective economic model predictive control for gas turbine system based on quantum simultaneous whale optimization algorithm. Energy Conversion and Management.

[ref-39] Huang F, Wang L, He Q (2007). An effective co-evolutionary differential evolution for constrained optimization. Applied Mathematics and Computation.

[ref-40] Iacca G, dos Santos Junior VC, Veloso de Melo V (2021). An improved Jaya optimization algorithm with Lévy flight. Expert Systems with Applications.

[ref-41] Jain L, Katarya R, Sachdeva S (2020). Opinion leader detection using whale optimization algorithm in online social network. Expert Systems with Applications.

[ref-42] Kaveh A, Dadras A (2017). A novel meta-heuristic optimization algorithm: thermal exchange optimization. Advances in Engineering Software.

[ref-43] Kennedy J, Eberhart R (1995). Particle swarm optimization.

[ref-44] Khalilpourazari S, Pasandideh SHR, Ghodratnama A (2018). Robust possibilistic programming for multi-item EOQ model with defective supply batches: whale optimization and water cycle algorithms. Neural Computing and Applications.

[ref-45] Liang JJ, Qu BY, Suganthan PN (2013). Problem definitions and evaluation criteria for the CEC 2014 special session and competition on single objective real-parameter numerical optimization.

[ref-46] Liu D, Fan Z, Fu Q, Li M, Faiz MA, Ali S, Li T, Zhang L, Khan MI (2020). Random forest regression evaluation model of regional flood disaster resilience based on the whale optimization algorithm. Journal of Cleaner Production.

[ref-47] Liu W, Wang Z, Yuan Y, Zeng N, Hone K, Liu X (2021). A novel sigmoid-function-based adaptive weighted particle swarm optimizer. IEEE Transactions on Cybernetics.

[ref-48] Liu M, Yao X, Li Y (2020). Hybrid whale optimization algorithm enhanced with Lévy flight and differential evolution for job shop scheduling problems. Applied Soft Computing.

[ref-49] Mafarja MM, Mirjalili S (2017). Hybrid whale optimization algorithm with simulated annealing for feature selection. Neurocomputing.

[ref-50] Mahdad B (2018). Improvement optimal power flow solution under loading margin stability using new partitioning whale algorithm. International Journal of Management Science and Engineering Management.

[ref-51] Mallipeddi R, Suganthan PN, Pan QK, Tasgetiren MF (2011). Differential evolution algorithm with ensemble of parameters and mutation strategies. Applied Soft Computing.

[ref-52] Mezura-Montes E, Coello CAC, Gelbukh A, de Albornoz A, Terashima-Marín H (2005). Useful infeasible solutions in engineering optimization with evolutionary algorithms. MICAI 2005: advances in artificial intelligence. MICAI 2005. Lecture notes in computer science, vol 3789.

[ref-53] Mezura-Montes E, Coello CAC (2008). An empirical study about the usefulness of evolution strategies to solve constrained optimization problems. International Journal of General Systems.

[ref-54] Mezura-Montes E, Hernández-Ocana B (2008). Bacterial foraging for engineering design problems: preliminary results.

[ref-55] Minh H-L, Sang-To T, Khatir S, Wahab MA, Cuong-Le T (2023a). Damage identification in high-rise concrete structures using a bio-inspired meta-heuristic optimization algorithm. Advances in Engineering Software.

[ref-56] Minh H-L, Sang-To T, Theraulaz G, Wahab MA, Cuong-Le T (2023b). Termite life cycle optimizer. Expert Systems with Applications.

[ref-57] Minh H-L, Sang-To T, Wahab MA, Cuong-Le T (2022). A new metaheuristic optimization based on K-means clustering algorithm and its application to structural damage identification. Knowledge-Based Systems.

[ref-58] Mirjalili S (2015). Moth-flame optimization algorithm: a novel nature-inspired heuristic paradigm. Knowledge-Based Systems.

[ref-59] Mirjalili S, Lewis A (2016). The whale optimization algorithm. Advances in Engineering Software.

[ref-60] Mirjalili S, Mirjalili SM, Lewis A (2014). Grey wolf optimizer. Advances in Engineering Software.

[ref-61] Mohammadi B, Mehdizadeh S (2020). Modeling daily reference evapotranspiration via a novel approach based on support vector regression coupled with whale optimization algorithm. Agricultural Water Management.

[ref-62] Montemurro M, Vincenti A, Vannucci P (2013). The automatic dynamic penalisation method (ADP) for handling constraints with genetic algorithms. Computer Methods in Applied Mechanics and Engineering.

[ref-63] Nazari-Heris M, Mehdinejad M, Mohammadi-Ivatloo B, Babamalek-Gharehpetian G (2017). Combined heat and power economic dispatch problem solution by implementation of whale optimization method. Neural Computing and Applications.

[ref-64] Ngo TT, Sadollah A, Kim JH (2016). A cooperative particle swarm optimizer with stochastic movements for computationally expensive numerical optimization problems. Journal of Computational Science.

[ref-65] Parsopoulos KE, Vrahatis MN (2005). Unified particle swarm optimization for solving constrained engineering optimization problems.

[ref-66] Pham Q-V, Mirjalili S, Kumar N, Alazab M, Hwang W-J (2020). Whale optimization algorithm with applications to resource allocation in wireless networks. IEEE Transactions on Vehicular Technology.

[ref-67] Qais MH, Hasanien HM, Alghuwainem S (2020a). Enhanced whale optimization algorithm for maximum power point tracking of variable-speed wind generators. Applied Soft Computing.

[ref-68] Qais MH, Hasanien HM, Alghuwainem S (2020b). Whale optimization algorithm-based Sugeno fuzzy logic controller for fault ride-through improvement of grid-connected variable speed wind generators. Engineering Applications of Artificial Intelligence.

[ref-69] Qiao Z, Shan W, Jiang N, Heidari AA, Chen H, Teng Y, Turabieh H, Mafarja M (2022). Gaussian bare-bones gradient-based optimization: towards mitigating the performance concerns. International Journal of Intelligent Systems.

[ref-70] Qiao W, Yang Z, Kang Z, Pan Z (2020). Short-term natural gas consumption prediction based on Volterra adaptive filter and improved whale optimization algorithm. Engineering Applications of Artificial Intelligence.

[ref-71] Ray T, Liew K-M (2003). Society and civilization: an optimization algorithm based on the simulation of social behavior. IEEE Transactions on Evolutionary Computation.

[ref-72] Reddy PDP, Reddy VCV, Manohar TG (2017). Whale optimization algorithm for optimal sizing of renewable resources for loss reduction in distribution systems. Renewables: Wind, Water, and Solar.

[ref-73] Rosyadi A, Penangsang O, Soeprijanto A (2017). Optimal filter placement and sizing in radial distribution system using whale optimization algorithm.

[ref-74] Saidala RK, Devarakonda N (2017). Improved whale optimization algorithm case study: clinical data of anaemic pregnant woman. Advances in Intelligent Systems and Computing.

[ref-75] Samadianfard S, Hashemi S, Kargar K, Izadyar M, Mostafaeipour A, Mosavi A, Nabipour N, Shamshirband S (2020). Wind speed prediction using a hybrid model of the multi-layer perceptron and whale optimization algorithm. Energy Reports.

[ref-76] Sang-To T, Hoang-Le M, Wahab MA, Cuong-Le T (2022). An efficient planet optimization algorithm for solving engineering problems. Scientific Reports.

[ref-77] Shadravan S, Naji HR, Bardsiri VK (2019). The sailfish optimizer: a novel nature-inspired metaheuristic algorithm for solving constrained engineering optimization problems. Engineering Applications of Artificial Intelligence.

[ref-78] Sreenu K, Sreelatha M (2017). W-Scheduler: whale optimization for task scheduling in cloud computing. Cluster Computing.

[ref-79] Srivastava A, Das DK, Rai A, Raj R (2018). Parameter estimation of a permanent magnet synchronous motor using whale optimization algorithm.

[ref-80] Storn R, Price K (1997). Differential evolution—a simple and efficient heuristic for global optimization over continuous spaces. Journal of Global Optimization.

[ref-81] Suganthan PN, Hansen N, Liang JJ, Deb K, Chen Y-P, Auger A, Tiwari S (2005). Problem definitions and evaluation criteria for the CEC 2005 special session on real-parameter optimization.

[ref-82] Talbi E-G (2009). Metaheuristics: from design to implementation.

[ref-83] Tran V-T, Nguyen T-K, Nguyen-Xuan H, Wahab MA (2023). Vibration and buckling optimization of functionally graded porous microplates using BCMO-ANN algorithm. Thin-Walled Structures.

[ref-84] Trivedi IN, Bhoye M, Bhesdadiya RH, Jangir P, Jangir N, Kumar A (2016). An emission constraint environment dispatch problem solution with microgrid using Whale Optimization Algorithm.

[ref-85] Tu J, Chen H, Liu J, Heidari AA, Zhang X, Wang M, Ruby R, Pham Q-V (2021). Evolutionary biogeography-based whale optimization methods with communication structure: towards measuring the balance. Knowledge-Based Systems.

[ref-86] Wang M, Chen H (2020). Chaotic multi-swarm whale optimizer boosted support vector machine for medical diagnosis. Applied Soft Computing.

[ref-87] Wang WL, Li WK, Wang Z, Li L (2019). Opposition-based multi-objective whale optimization algorithm with global grid ranking. Neurocomputing.

[ref-88] Wang H, Rahnamayan S, Sun H, Omran MGH (2013). Gaussian bare-bones differential evolution. IEEE Transactions on Cybernetics.

[ref-89] Wang H, Rahnamayan S, Wu Z (2013). Parallel differential evolution with self-adapting control parameters and generalized opposition-based learning for solving high-dimensional optimization problems. Journal of Parallel and Distributed Computing.

[ref-90] Wolpert DHH, Macready WGG (1997). No free lunch theorems for optimization. IEEE Transactions on Evolutionary Computation.

[ref-91] Wu J, Wang H, Li N, Yao P, Huang Y, Yang H (2018). Path planning for solar-powered UAV in urban environment. Neurocomputing.

[ref-92] Yuan P, Guo C, Zheng Q, Ding J (2018). Sidelobe suppression with constraint for MIMO radar via chaotic whale optimisation. Electronics Letters.

[ref-93] Zeng N, Song D, Li H, You Y, Liu Y, Alsaadi FE (2021). A competitive mechanism integrated multi-objective whale optimization algorithm with differential evolution. Neurocomputing.

[ref-94] Zhang Z (2015). A new multi-population-based differential evolution. International Journal of Computing Science and Mathematics.

[ref-95] Zhang J, Sanderson AC (2009). JADE: adaptive differential evolution with optional external archive. IEEE Transactions on Evolutionary Computation.

[ref-96] Zhang J, Xiao M, Gao L, Pan Q (2018). Queuing search algorithm: a novel metaheuristic algorithm for solving engineering optimization problems. Applied Mathematical Modelling.

[ref-97] Zhao W, Wang L, Zhang Z (2019). Supply-demand-based optimization: a novel economics-inspired algorithm for global optimization. IEEE Access.

[ref-98] Zhu W, Tang Y, Fang J, Zhang W (2013). Adaptive population tuning scheme for differential evolution. Information Sciences.

[ref-99] Zou F, Chen D, Lu R, Wang P (2017). Hierarchical multi-swarm cooperative teaching—learning-based optimization for global optimization. Soft Computing.

